# Catchment-Scale Conservation Units Identified for the Threatened Yarra Pygmy Perch (*Nannoperca obscura*) in Highly Modified River Systems

**DOI:** 10.1371/journal.pone.0082953

**Published:** 2013-12-13

**Authors:** Chris J. Brauer, Peter J. Unmack, Michael P. Hammer, Mark Adams, Luciano B. Beheregaray

**Affiliations:** 1 Molecular Ecology Laboratory, School of Biological Sciences, Flinders University, Adelaide, South Australia, Australia; 2 Institute for Applied Ecology and Collaborative Research Network for Murray-Darling Basin Futures, University of Canberra, Canberra, Australian Capital Territory, Australia; 3 School of Earth and Environmental Sciences, University of Adelaide, South Australia, Australia; 4 Curator of Fishes, Museum and Art Gallery of the Northern Territory, Darwin, Northern Territory, Australia; 5 Evolutionary Biology Unit, South Australian Museum, Adelaide, South Australia, Australia; School of Environment & Life Sciences, United Kingdom

## Abstract

Habitat fragmentation caused by human activities alters metapopulation dynamics and decreases biological connectivity through reduced migration and gene flow, leading to lowered levels of population genetic diversity and to local extinctions. The threatened Yarra pygmy perch, *Nannoperca obscura*, is a poor disperser found in small, isolated populations in wetlands and streams of southeastern Australia. Modifications to natural flow regimes in anthropogenically-impacted river systems have recently reduced the amount of habitat for this species and likely further limited its opportunity to disperse. We employed highly resolving microsatellite DNA markers to assess genetic variation, population structure and the spatial scale that dispersal takes place across the distribution of this freshwater fish and used this information to identify conservation units for management. The levels of genetic variation found for *N. obscura* are amongst the lowest reported for a fish species (mean heterozygosity of 0.318 and mean allelic richness of 1.92). We identified very strong population genetic structure, nil to little evidence of recent migration among demes and a minimum of 11 units for conservation management, hierarchically nested within four major genetic lineages. A combination of spatial analytical methods revealed hierarchical genetic structure corresponding with catchment boundaries and also demonstrated significant isolation by riverine distance. Our findings have implications for the national recovery plan of this species by demonstrating that *N. obscura* populations should be managed at a catchment level and highlighting the need to restore habitat and avoid further alteration of the natural hydrology.

## Introduction

Human activities such as land development, agriculture, and exploitation of natural resources have long been acknowledged as driving processes behind habitat fragmentation and degradation [1], [2]. This can decrease population connectivity through reduced migration and gene flow, leading to higher genetic differentiation among populations and lowered levels of genetic diversity within [3]. When populations become isolated they become vulnerable to extirpation due to environmental [1], demographic [4] and genetic [5] processes that increase the chances of local extinction. If habitat fragmentation is widespread on a regional scale then there is the potential for loss of biodiversity and species extinctions [6]. It is therefore important for conservation and natural resource managers to consider patterns and processes related to population connectivity and gene flow at both local and regional scales [7].

Conservation genetics is the application of evolutionary principles and molecular methods to species and biodiversity conservation [8]. Riverscapes have long been recognised for their ecological complexity and sensitivity to human impacts [9], [10], and have been the focus of many recent conservation genetics studies [11]–[15]. Understanding the spatial scale of patterns of genetic diversity is also important for species conservation in order to identify the evolutionary processes shaping these patterns and to detect when populations become demographically and genetically independent [16], [17]. This information can be used to estimate the geographical extent of conservation units defined by genetic criteria [18], [19], such as the popularly used Evolutionarily Significant Units (ESUs) and Management Units (MUs) (see Moritz [20] and Crandall [21] for definitions). These conservation units can inform conservation management strategies by recognising the historical isolation of evolutionary lineages (i.e. ESU), and the functional and demographic independence of populations (i.e. MU) [22].

**Figure 1:**
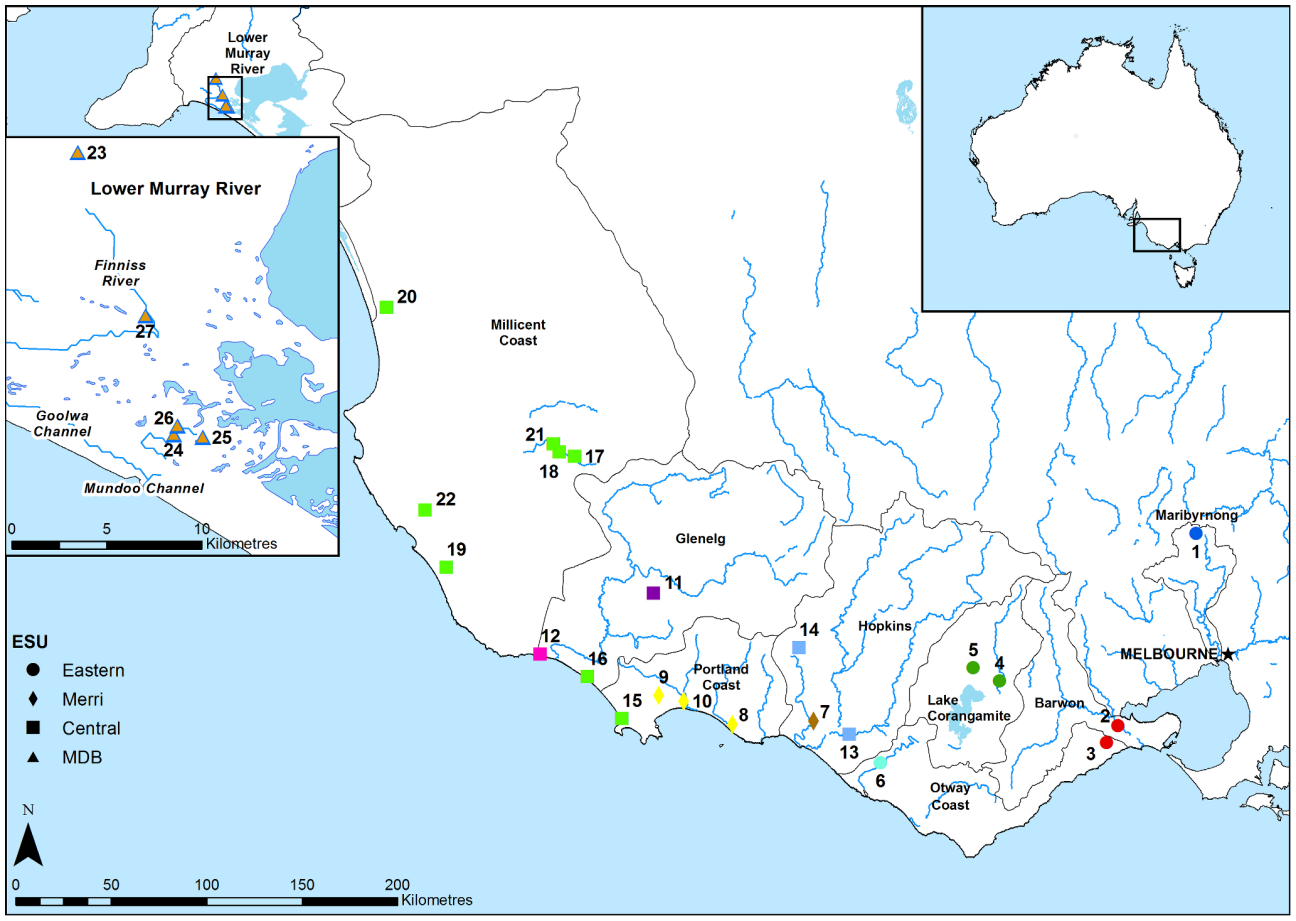
*Nannoperca obscura* sampling locations and proposed Evolutionarily Significant Units (ESUs). Inset shows close-up of the MDB sites. Colours denote genetic clusters (MUs) described in Figure 2.

In this study we identify units for conservation in a threatened freshwater fish, *Nannoperca obscura* (Yarra pygmy perch), found across two biogeographic provinces [23] in a series of adjacent, but highly fragmented Australian riverine ecosystems. One is the Murray-Darling Basin (MDB), arguably Australia's most important agricultural region, given it contributes 50% of the water used for agricultural irrigation in the country [24]. Modifications to the natural flow regime, water abstraction, drainage of wetlands, and the introduction of exotic species have all contributed to the decline of native fishes across the MDB, and this trend is expected to continue in the face of future climate change [25]. In recent years drought has led to extremely low inflows and reduced water levels throughout the system and this is especially evident in the Lower Lakes region of the MDB [26], [27]. Bass Province is the second biogeographically distinct area which contains many smaller separate catchments encompassing most of the range of *N. obscura* (Fig. 1). Most drainages have experienced considerable alterations due to major agricultural activity and as a result the region contains some of the most highly disturbed waterways in Australia [28]. The extensive alterations to the natural surface water hydrology here have had a major impact, resulting in wetland drainage and reduction in freshwater habitat [29].

*Nannoperca obscura* (family Percichthyidae) is a small (up to 75 mm total length) freshwater fish endemic to coastal drainages of southeastern Australia [30] (Fig. 1). This species prefers slow flowing habitat along the edge of streams and rivers, with abundant submerged vegetation [31]. They are largely sedentary, with large demersal larvae suggesting limited dispersal potential at all life stages [32]. *Nannoperca obscura* has declined since European settlement, and is currently listed as vulnerable by the IUCN [33], protected and critically endangered in South Australia [34], vulnerable in Victoria [35], and has a National Species Recovery Plan [36]. Habitat fragmentation has been exacerbated by recent drought and is considered a major threat along with competition and predation by introduced species and climate change [30]. The decline of *N. obscura* in the MDB and southeastern South Australia was recently exacerbated by extreme drought, which resulted in the extirpation of all populations in the lower Murray River [37]. However, remnants of these populations were rescued before their extirpation and form the basis for a conservation breeding program, with offspring from this effort being released back into former habitats since drought conditions eased in 2011 [27]. In order to guide conservation efforts of *N. obscura*, Hammer et al. [32] conducted a phylogeographic study using mtDNA and allozymes. They identified four evolutionary lineages as ESUs, which they designated Eastern, Merri, Central, and MDB, and tentatively suggested that most catchments contained separate MUs.

Here we employed recently developed microsatellite DNA markers to provide finer resolution of recent genetic divergences. This, combined with greatly increased sampling, allows a more detailed assessment of population structure and genetic diversity over multiple spatial scales (i.e. within and between catchments) across the entire distribution of *N. obscura*. In order to address issues of relatedness between isolated river systems, we treat each river with an independent connection to the ocean as a separate unit, herein defined as a catchment. Firstly, we used these data to test the proposed ESUs of Hammer et al. [32], which were defined with more conservative molecular markers using a relatively small sample of individuals (n = 156) and localities (18). Secondly, we tested the hypothesis derived from life history and ecological requirements that *N. obscura* are poor dispersers and should therefore display very low connectivity between catchments and low connectivity between sites within catchments, with many populations representing different MUs within highly structured ESUs. For this hypothesis, we use a combination of frequency-based and genotypic-based statistical methods to assess the number and spatial distribution of MUs within inferred ESUs. Finally, we explore factors that have shaped the spatial distribution of genetic diversity in *N. obscura* and highlight the direct implications of our findings for conservation management.

## Materials and Methods

### Ethics Statement

Permission to undertake field work and collect specimens was obtained under the following permits: Victorian Fisheries research permits RP 581 and RP 945, Victorian Flora and Fauna permits 10002072 and 10004939, Victorian National Parks permit 10004939, South Australian Primary Industries and Resources - Section 59 and 115 Exemptions. Specimens were obtained under Arizona State University Institutional Animal Care and Use Committee (IACUC) approval 09-1018R, Brigham Young University IACUC approval 070403, University of Adelaide Animal Ethics Committee approval S-32-2002 and Flinders University Animal Welfare Committee approval E313.

### Sampling and genotyping

A total of 541 individuals were sampled from 27 locations (n = 5–40 per site) across the entire extant range of *N. obscura* (Fig. 1). DNA was extracted from caudal fin clips following a modified salting out process [38]. Fourteen microsatellite loci designed specifically for *N. obscura* were amplified in two polymerase chain reaction (PCR) multiplexes of six and eight loci, respectively [39] (Table S1). The PCR conditions were based on a modified touchdown procedure [40]. The PCR product was diluted 1∶5 with H_2_O, sized with GS500LIZ size standard and analysed using an automated ABI 3130 capillary electrophoresis system (Applied Biosystems) with one run per multiplex PCR. Genotypes were binned and scored visually with GeneMapper 4.0 (Applied Biosystems). Genotypes were checked for scoring errors putatively related to null alleles, stuttering, and large allele drop-out using Micro-Checker 2.2.3 [41]. To ensure all loci were scored consistently, we repeated amplification and genotyping procedures for 86 individuals.

### Genetic variation

Fisher's exact test of linkage disequilibrium and tests for departures from Hardy-Weinberg equilibrium (HWE) were conducted using GENEPOP 4.1.4 [42], and GenoDive 2.0 [43], respectively. Significance levels were Bonferroni-corrected to avoid type I errors associated with multiple tests [44]. For each site, the number of alleles (N_A_), expected (H_E_) and observed (H_O_) heterozygosity, and inbreeding coefficient (F_IS_) were calculated in GenoDive 2.0 [43]. Allelic richness (A_R_) was estimated using the rarefaction procedure in HP-RARE [45], and the percentage of polymorphic loci was calculated in GenAlEx 6.5 [46].

### Population structure

Population genetic structure was assessed at multiple spatial scales, both across the entire distribution and within each of the proposed ESUs, using a combination of several frequency-based and genotype-based statistical methods. Pairwise F_ST_ [47] and R_ST_ [48] tests were performed in Arlequin 3.5.1.2 [49] to evaluate between-site differentiation. Given the potential for temporal variation in population structure, an assessment of pairwise F_ST_ and R_ST_ was also performed between years at 12 sites where samples were collected on multiple occasions. In order to determine if either F_ST_ or R_ST_ was more appropriate for this study, the relative contribution of genetic drift and mutation to population differentiation was assessed [50]. SPAGeDi 1.3 [51] was used to permutate global allele sizes for each locus and to compare observed R_ST_ with permutated R_ST_ (pR_ST_) values. Arlequin was used to perform an analysis of molecular variance (AMOVA) with 1000 permutations based on F_ST_ [47]. Hierarchical structure was assessed using AMOVA among the major genetic lineages, among sites within lineages, and among individuals within sites. Separate AMOVAs were also performed for each primary lineage.

A Bayesian clustering analysis of individual genotypes using STRUCTURE 2.3.4 [52] was initially performed using all samples to identify primary population structure across the entire distribution, before repeating the analysis within each of the primary clusters to assess hierarchical population structure at smaller spatial scales [53]. Twenty independent runs for each K value (1–27) were completed to ensure reproducibility [54], using a burn in of 100 000 followed by 1 million Monte-Carlo Markov chain (MCMC) iterations. We used the admixture model, with independent allele frequencies among populations and no prior information on sampling location. The most likely K value was inferred using the Evanno et al. method [53] implemented in STRUCTURE HARVESTER [55]. Results of the 20 replications were then combined using the software CLUMPP 1.1.2 [56], and visualised using Distruct 1.1 [57]. A different analytical approach based on assignment of individual genotypes was performed using GeneClass2 [58]. This was conducted using the Bayesian approach of Rannala and Mountain [59] to calculate the probability that each individual originates from its sampling locality or from other sites.

Principal coordinates analysis (PCA) was also employed to allow visual examination of the genetic affinities of individuals across the entire distribution and to clusters identified within each lineage. Pairwise genetic distances [60] between individuals were first calculated before those results were subjected to PCA analysis. Both procedures were completed in GenAlEx 6.5 [46].

### Gene flow

We used BayesAss 3.0 [61] to estimate recent migration among lineages and also between clusters identified within lineages. BayesAss implements a Bayesian MCMC resampling method using multilocus genotypes to estimate asymmetrical rates of recent migration, where migration (*m*) is the proportion of each population having migrant ancestry. First generation migrants, or the offspring of at least one first generation migrant, are considered as having migrant ancestry. The software was run for 10 million iterations with a 1 million iteration burn in. Mixing parameters for allele frequencies, inbreeding coefficients and migration rate were adjusted to achieve optimum acceptance rates of 20–40% [61]. Convergence was confirmed by plotting the cumulative log likelihoods of the iterations using the program Tracer 1.5 [62]. Each run was also repeated five times using different seeds and the posterior estimates compared for consistency [61].

### Spatial analyses

Spatial genetic structure was assessed at both the population and individual level. At the population level isolation by distance (IBD) was assessed using Mantel tests. These tests were applied to each of the three lineages that displayed evidence of population structure to determine the association between pairwise population F_ST_ and geographic distance. Euclidean distance was calculated because most sites occur in isolated catchments and are not connected by continuous stream lengths.

Results of a test for IBD can be difficult to interpret when population structure is strongly influenced by sharply divided spatial groups [63]. This can occur when hierarchical structure due to the presence of strong barriers for dispersal (physical or ecological) creates clusters of populations that are not necessarily better explained by spatial distances between demes. To assess this possibility partial Mantel tests were performed to assess for hierarchical population structure, such as that identified by the AMOVAs and STRUCTURE. These tests assessed the association between F_ST_ and geographic distance while controlling for hierarchical population structure by using a binary model matrix describing whether comparisons were made either between or within the identified clusters [64]. Finally a simple Mantel test was performed for C4, the largest genetic cluster identified within a lineage (no other clusters contained a sufficient number of sites), in order to test for IBD at a local scale. Performing this test separately within predefined clusters removes any potential bias of hierarchical structure [63]. All Mantel and partial Mantel tests were performed in GenoDive 2.0 using 1000 permutations.

To further evaluate spatial dimensions of genetic structure at an individual level within each lineage we used spatial autocorrelation [65] in two ways. Firstly, correlograms were constructed using the method of Smouse and Peakall [60] implemented in GenAlEx 6.5. The autocorrelation coefficient (*r*) was plotted as a function of discrete distance classes, partitioned so as to achieve a similar number of pairwise comparisons for each class [18]. A positive *r* value indicates the presence of IBD and the *x* intercept can provide an estimate of the extent of IBD for each lineage [17]–[19]. Peakall et al. [17] also suggest a second autocorrelation method to accurately identify the scale at which population genetic structure is detectable. In this case, *r* was calculated using multiple distance class analysis, also in GenAlEx 6.5. This method plots *r* as a function of increasing distance class sizes [17]. The first class is based on the minimum distance between sites (0–10 km for all lineages) and each successive class adds individuals from more distant groups (i.e. 0–10 km, 0–20 km, 0–30 km, etc.). When significant IBD exists, the value of *r* is expected to decrease with the increasing size of each distance class. The last distance class for which *r* is significant is considered the limit of detectable IBD [17]. Significance was assessed for both tests using 95% confidence intervals for the null hypothesis of no spatial structure using 999 random permutations, and for estimates of *r* by bootstrapping 1000 pairwise comparisons for each distance class [46].

## Results

### Data quality and genetic variation

There was no consistent evidence for stuttering or large allele drop-out for any locus, or for linkage disequilibrium between any pairs of loci. Null alleles were detected for Nob26 at sites 14 and 26, Nob30 at sites 2, 5 and 17 and Nob35 at site 27. However as these findings were not consistent across populations, and when analyses were run without these loci similar results were obtained, all loci were retained. After Bonferroni correction, only one sampled site (#2, Waurn Ponds Creek) was found to deviate significantly from expectations of Hardy-Weinberg equilibrium (Table 1). The 14 microsatellite loci contained between 4 and 26 alleles, with a mean of 11.9 alleles per locus. Despite the highly polymorphic nature of the markers used, the overall levels of genetic variation were very low for *N. obscura*, with mean observed heterozygosity of 0.318 and mean allelic richness of 1.92 (Table 1). No major differences in genetic variation were apparent between sampled sites or among putatively different lineages. All repeated individuals generated the same genotype.

**Table 1:** Information on localities, sample sizes, Evolutionarily Significant Units (ESUs), Management Units (MUs) and summary of genetic diversity for *Nannoperca obscura*.

Site	ESU	MU	Location	Latitude	Longitude	N	N_A_	% Poly loci	A_R_	H_O_	H_E_	F_IS_	P value
1	Eastern	E1	Deep Ck, Lancefield	−37.259	144.713	10	1.9	43%	1.41	0.150	0.149	−0.005	0.504
2		E2	Waurn Ponds Ck, Geelong	−38.189	144.349	33	4.0	93%	2.41	0.468	0.521	0.102	**0.001**
3		E2	Thompson Ck	−38.272	144.290	10	2.9	86%	2.16	0.386	0.424	0.090	0.126
4		E3	Woady Yaloak R, Cressy	−38.024	143.627	7	2.0	64%	1.80	0.347	0.346	−0.002	0.476
5		E3	Gnarkeet Ck, Lismore	−37.972	143.466	29	2.1	57%	1.66	0.241	0.266	0.093	0.064
6*		M1	Curdies R, Curdie	−38.448	142.957	30	2.5	79%	1.77	0.277	0.304	0.090	0.032
7	Merri	M2	Merri R, Grassmere	−38.275	142.542	39	3.7	71%	2.02	0.364	0.377	0.033	0.199
8		M3	Shaw R, Yambuk	−38.315	142.061	40	4.7	79%	2.22	0.378	0.391	0.034	0.147
9		M3	Surry R, Heathmere	−38.200	141.614	30	3.9	93%	2.36	0.452	0.438	−0.032	0.198
10		M3	Fitzroy R, Tyrendarra	−38.221	141.764	8	3.4	79%	2.45	0.390	0.462	0.155	0.014
11	Central	C1	Palmer Ck, Merino	−37.724	141.546	31	2.7	71%	1.94	0.320	0.343	0.066	0.063
12		C2	Crescent Pond, Picks Swamp	−38.040	140.898	20	2.1	86%	1.39	0.136	0.162	0.160	0.030
13		C3	Mount Emu Ck, Panmure	−38.325	142.759	31	2.4	79%	1.56	0.209	0.224	0.066	0.119
14		C3	Mustons Ck	−37.936	142.427	8	2.8	86%	2.10	0.357	0.391	0.086	0.144
15		C4	Bridgewater Lakes, main lake	−38.319	141.405	10	3.0	86%	2.21	0.414	0.425	0.025	0.368
16		C4	Lake Monibeong	−38.133	141.186	10	2.8	93%	2.10	0.407	0.427	0.047	0.308
17		C4	Mosquito Ck, Langkoop	−37.104	141.037	34	2.6	86%	1.80	0.290	0.314	0.077	0.036
18		C4	Mosquito Ck, Wombeena	−37.087	140.945	10	2.3	71%	1.83	0.257	0.333	0.227	0.006
19		C4	Drain 88, Lake Bonney	−37.657	140.316	9	2.3	86%	1.85	0.278	0.330	0.158	0.039
20		C4	Henry Ck, Kingston	−36.450	139.891	16	3.0	86%	1.95	0.366	0.360	−0.018	0.446
21		C4	Mosquito Ck, South Waverley	−37.052	140.908	14	2.2	79%	1.78	0.280	0.319	0.121	0.047
22		C4	Drain M, Elgin Lane	−37.393	140.174	26	2.6	86%	1.91	0.323	0.359	0.100	0.022
23	MDB	MDB	Finniss R, L. Alexandrina	−35.405	138.843	5	2.2	64%	1.90	0.300	0.323	0.072	0.241
24		MDB	Mundoo Channel, Hindmarsh Is.	−35.537	138.905	5	2.0	64%	1.74	0.300	0.273	−0.098	0.238
25		MDB	Mundoo Channel, Hindmarsh Is.	−35.538	138.922	27	3.1	79%	1.85	0.302	0.313	0.037	0.241
26		MDB	Steamer Drain, Hindmarsh Is.	−35.533	138.907	17	2.3	71%	1.82	0.291	0.302	0.037	0.270
27		MDB	Goolwa Ch. Lake Alexandrina	−35.481	138.886	32	2.9	79%	1.82	0.293	0.297	0.015	0.359

_R_ is allelic richness, H_O_ is observed heterozygosity, H_E_ is expected heterozygosity, F_IS_ is inbreeding coefficient, P value relates to Hardy-Weinberg equilibrium test (significant value indicated in bold). N is number of samples, A

Site 6 (Curdies River) is an Eastern ESU site with high levels of admixture from Merri and as such has been included in Merri for all analyses.

### Population structure

**Figure 2:**
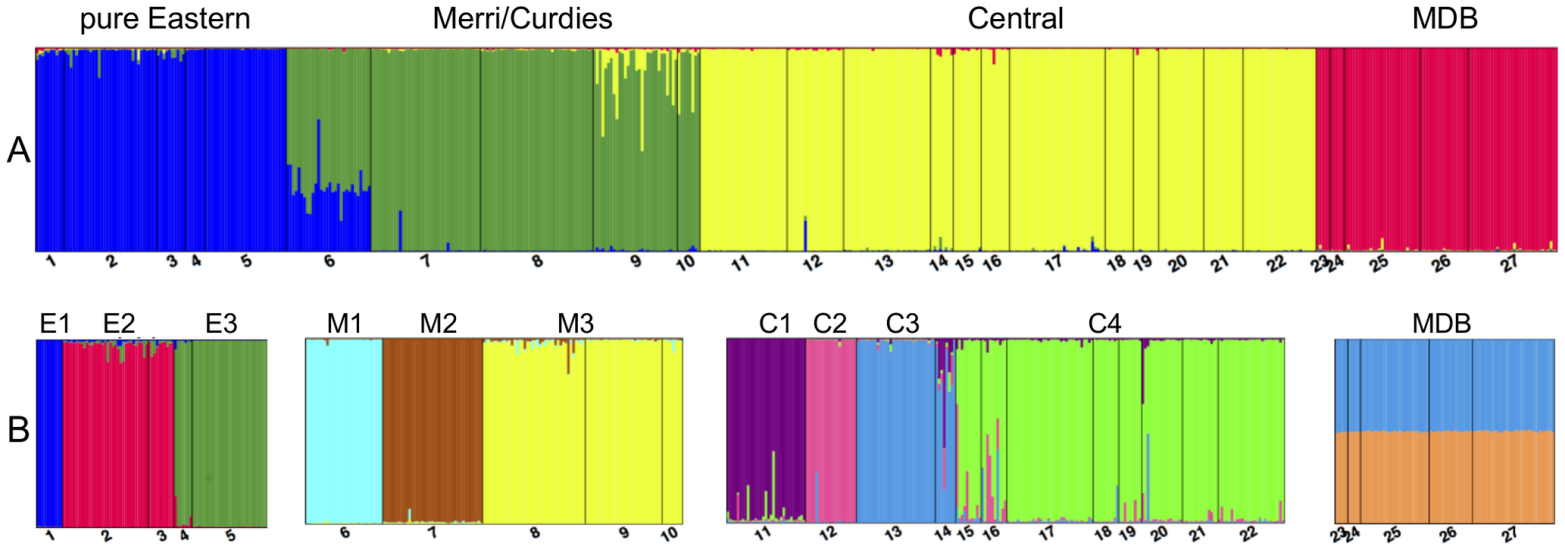
Admixture plots indicating major genetic lineages and Management Units (MUs) for *Nannoperca obscura* produced by the software STRUCTURE. A) K = 4 selected as most likely K value by STRUCTURE HARVESTER, and B) hierachical structure indicating proposed MUs. Site numbers correspond to those in Table 1 and Fig. 1.

Clustering analysis in STRUCTURE demonstrated high levels of differentiation, identifying four major clusters (Fig. 2A). These generally correspond with the proposed ESUs of Hammer et al. [32], although our microsatellite data displayed evidence of significant admixture between the Eastern and Merri ESUs at site 6 (Curdies River, Eastern ESU; Hammer et al. [32]), to the extent that site 6 was assigned to the Merri cluster. Since contemporary genetic and demographic processes are the focus of the present study, Curdies River was included as part of the Merri genetic lineage for all analyses. Apart from the lack of differentiation within the MDB, strong population structure was evident within the other lineages. Three genetic clusters were identified within the ‘pure Eastern’ lineage, three within Merri/Curdies, and four within the Central lineage (Fig. 2B). Population assignment results from GeneClass2 strongly support the population structure identified by STRUCTURE (Table S2). Most individuals (66.9%) were correctly assigned to their sampling location, with very few assigned to sites outside of their proposed lineage for probabilities greater than 5%. Within the four primary genetic lineages, sites that shared a high probability of assignment closely correspond to the genetic clusters identified by STRUCTURE.

As predicted by the STRUCTURE analyses, significant and very high levels of genetic structure were evident between most demes of *N. obscura* (341 pairwise comparisons were significant out of 351 tests), with F_ST_ ranging from 0–0.84 (mean F_ST_ = 0.45), and R_ST_ ranging from 0–0.95 (mean R_ST_ = 0.49) (Table S3). Assessment of temporal variation in allele frequencies at sites sampled on multiple years did not reveal any temporal trend in population structure, with only one statistically significant comparison out of 24 pairwise tests (Table S4). The comparison of R_ST_ and pR_ST_ in SPAGeDi revealed that pR_ST_ was significantly greater than R_ST_ for only two loci (Nob2; Nob12). This indicates that, for this dataset, genetic drift contributes more to genetic diversity than mutation. Therefore, F_ST_ was used as the measure of population differentiation [51].

Based on F_ST_, AMOVA calculated across all sites attributed 34% of the variation to differences among proposed primary lineages (P<0.001), 18% to variation between sites within lineages (P<0.001), and just 2.6% among individuals within sites (P<0.001) (Table 2). When calculated separately, the AMOVA results were similar for each of the pure Eastern, Merri/Curdies, and Central lineages, with ∼30% of the variation attributed to among site differences, and among individuals within sites only contributing 0.8% (P = 0.281) for Merri/Curdies, and 5.9% (P<0.001) and 6.3% (P<0.001) for Central and pure Eastern lineages, respectively (Table 2). No significant variation was detected among sites or among individuals within sites in the MDB (Table 2).

**Table 2:** Hierarchical analysis of molecular variance (AMOVA) based on F_ST_ for *Nannoperca obscura*.

Group	Source of variation	d. f.	% of variance	P value
All sites				
	Among lineages	3	34.9%	**<0.001**
	Among sites within lineages	23	18.2%	**<0.001**
	Among individuals within sites	514	2.6%	**<0.001**
pure Eastern				
	Among sites	4	29.7%	**<0.001**
	Among individuals within sites	84	6.3%	**<0.001**
Merri/Curdies				
	Among sites	4	30.6%	**<0.001**
	Among individuals within sites	142	0.8%	0.281
Central				
	Among sites	11	32.2%	**<0.001**
	Among individuals within sites	207	5.9%	**<0.001**
MDB				
	Among sites	4	2.5%	0.131
	Among individuals within sites	81	2.7%	0.200

Significant values indicated in bold.

**Figure 3:**
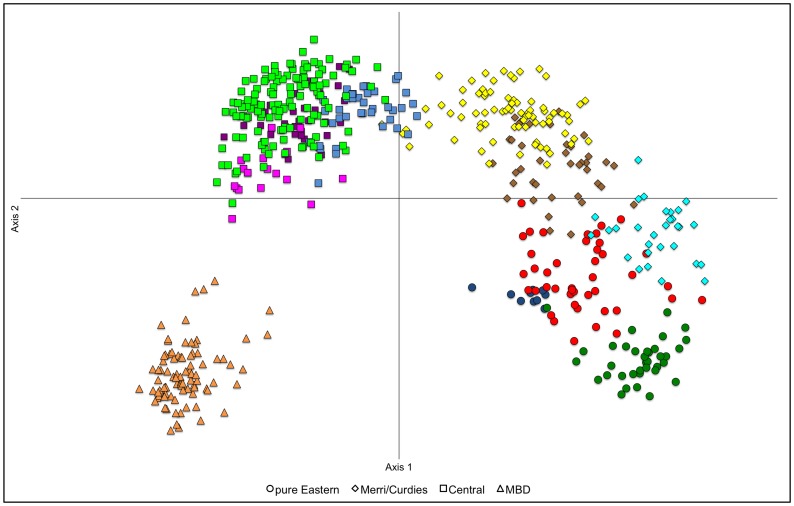
Principal coordinates analysis based on 14 microsatellite loci for *Nannoperca obscura* supporting the delineation of the four genetic lineages. Eigen values for the first and second axes have been plotted, which explain 35% and 25% of the variance, respectively. Colours denote genetic clusters (MUs) described in Figure 2.

The PCA results were also generally concordant with the other analyses of geographic population structure. The four primary genetic lineages were well supported in the initial analysis of all individuals (Fig. 3), while the genetic clusters identified within primary lineages (Table 1; Fig. 2B) were mostly supported when each was run separately (Fig. S1). Importantly, as also demonstrated by all other analyses, the PCA plot for MBD provided no evidence for population structure within this lineage (Fig. S1D).

### Gene flow

Estimates of recent gene flow in BayesAss demonstrate extremely low exchange of migrants both among lineages (0.2–0.7%) (Table S5) and between clusters within each primary genetic lineage (0.2–2.1%) (Table S6). All pairwise estimates of *m* were within the 95% credible interval and there was no evidence for asymmetric gene flow between populations. These results validate the delineation of genetically and demographically isolated clusters within the four lineages, and are in line with expectations given the high level of population structure indicated by the other analyses.

### Spatial analyses

**Figure 4:**
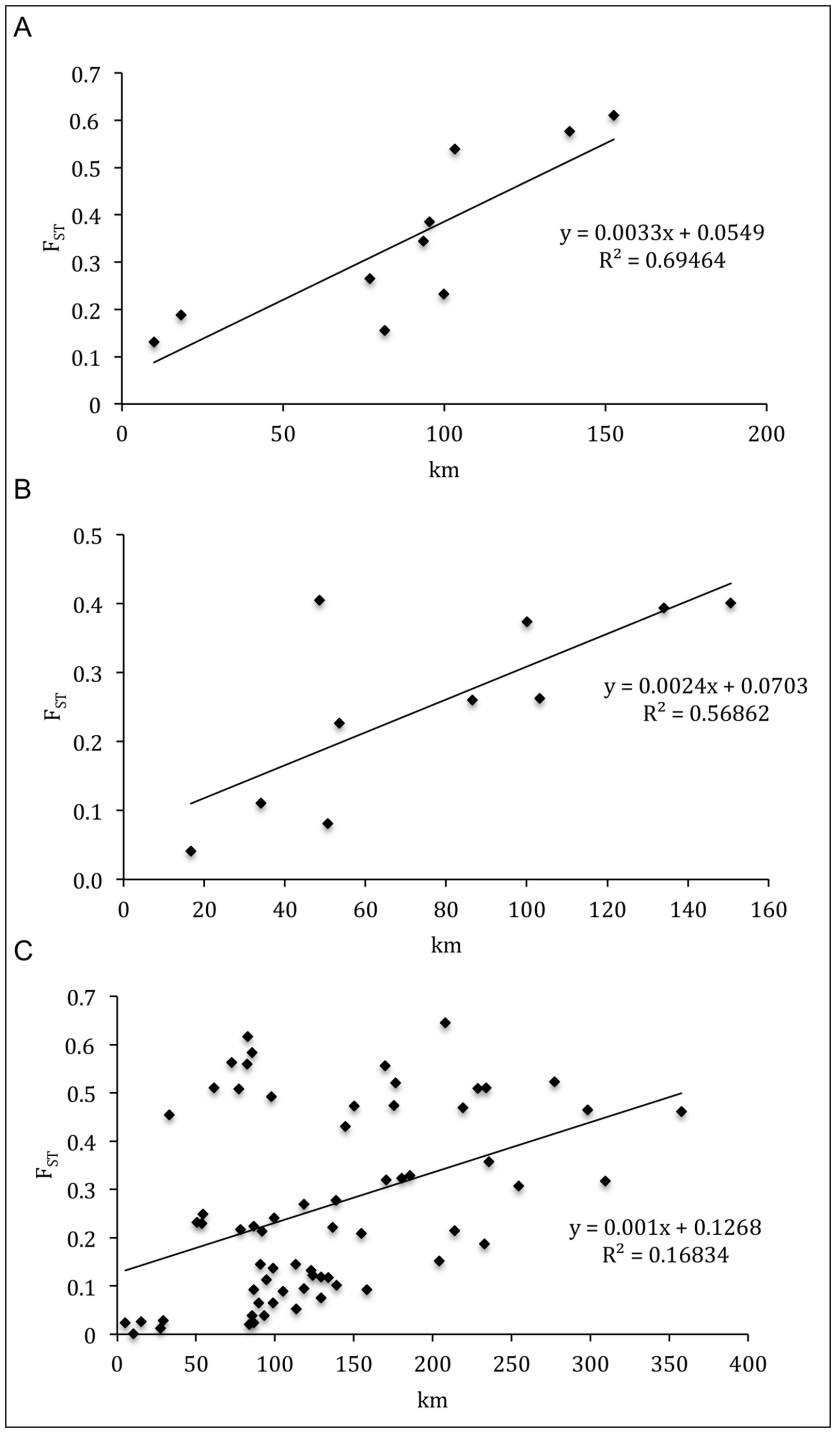
Isolation by distance plots for *Nannoperca obscura* comparing F_ST_ (Weir and Cockerham 1984) with distance between sites within A) pure Eastern, B) Merri/Curdies, and C) Central lineages.

Patterns of IBD and hierarchical population structure were revealed at multiple scales for *N. obscura*. Mantel tests demonstrated a strong, significant association between population F_ST_ and geographic distance (*r* values ranged between 0.411 and 0.833) (Table 3), indicating the presence of IBD between sites within the pure Eastern, Merri/Curdies, and Central lineages (Fig. 4). The results of the partial Mantel tests were not significant for Merri/Curdies (*r* = 0.43, P = 0.192) and Central (*r* = 0.18, P = 0.219) but were significant for the pure Eastern lineage (*r* = 0.0, P = 0.005) (Table 3). Although these tests suggest that hierarchical population structure due to catchment divisions is not evident within two lineages, they suffer from relatively low power due to the general lack of multiple samples representing each catchment. Finally, the strong and highly significant results of the Mantel test within the C4 genetic cluster (*r* = 0.785, P = 0.001) demonstrate the pattern of IBD also exists at a smaller scale for *N. obscura* (Table 3).

**Table 3:** Results for Mantel and partial Mantel tests for major genetic lineages, and genetic cluster C4 for *Nannoperca obscura*.

Lineage	Matrix A	Matrix B	Covariate	Mantel's r	P value
pure Eastern	Genetic	Geographic	–	0.833	**0.014**
	Genetic	Geographic	Clusters	0.900	**0.005**
erri/Curdies	Genetic	Geographic	–	0.754	**0.018**
	Genetic	Geographic	Clusters	0.431	0.192
entral	Genetic	Geographic	–	0.411	**0.030**
	Genetic	Geographic	Clusters	0.181	0.219
Cluster					
4	Genetic	Geographic	–	0.785	**0.001**

_ST_, geographic distance is Euclidean distance between sites (km). Significant values indicated in bold. Genetic distance is F

**Figure 5:**
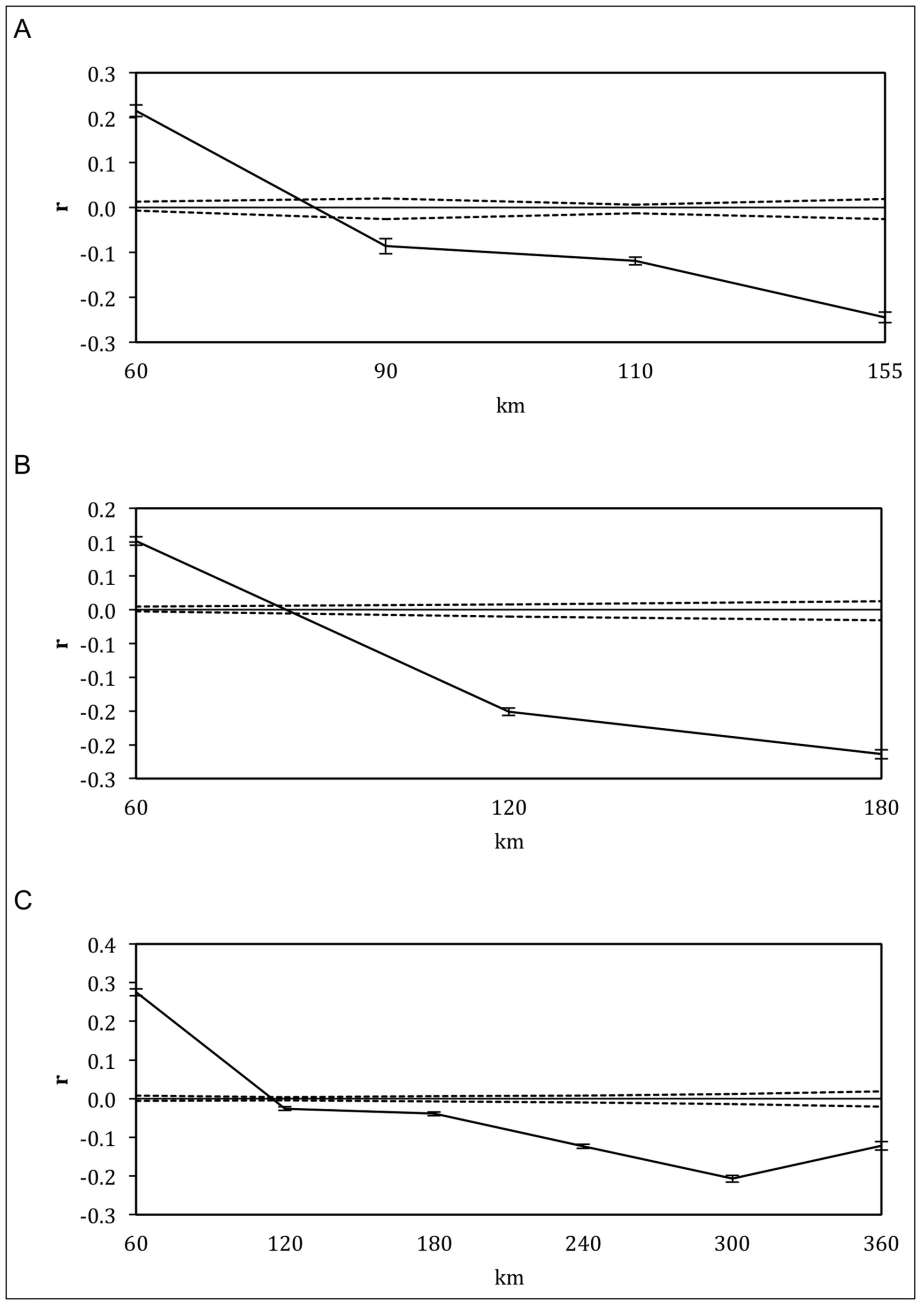
Correlograms showing the autocorrelation coefficient *r* as a function of distance for A) pure Eastern, B) Merri/Curdies, and C) Central lineages. Distances are the maximum for each class, dashed lines are the 95% CI about the null hypothesis of a random distribution of genotypes, and error bars are 95% confidence of *r*. Intercept values are 81 km, 84 km, and 115 km for A, B, and C respectively.

At the individual level, there was significant positive spatial autocorrelation for the first distance class (0–60 km), which intercepted the *x*-axes at 81 km, 84 km, and 115 km for the pure Eastern, Merri/Curdies, and Central correlograms, respectively (Fig. 5). This strongly indicates that on average, individuals from each locality had a higher probability of being born locally, providing support to the IBD signal demonstrated by the Mantel tests. To determine the extent to which this pattern of IBD exists, the autocorrelation coefficient *r* was also calculated for increasing distance class sizes. The positive *r* values became non-significant at 150 km for both pure Eastern and Merri/Curdies lineages (Fig. S2) and at 300 km for the putatively more connected Central lineage (Fig. S3). Significant IBD is therefore confirmed within the pure Eastern and Merri/Curdies lineages for sites up to 140 km apart, beyond which positive spatial autocorrelation is no longer detectable. The results are similar for the Central lineage, where IBD can be detected for sites up to 290 km apart.

## Discussion

We employed highly resolving microsatellite markers to assess genetic variation and population structure across the distribution of a threatened freshwater fish, *N. obscura*. Remarkably strong population genetic structure that corresponds with catchment boundaries was detected in a pattern that broadly validates the four ESUs previously proposed for this species [32]. Spatial analysis of genetic variation demonstrated that both significant IBD and hierarchical population structure exist in *N. obscura*. Several MUs were identified within each of the pure Eastern (3 MUs), Merri/Curdies (3 MUs), and Central (4 MUs) lineages (Table 1; Fig. 2B). The MDB however appears to contain a single genetic population which is consistent with the close geographic proximity of sampled sites (Fig. 1). A combination of spatial analytical methods was also implemented to determine the scale and extent of IBD within each ESU. Here we describe the spatial context of the strong subdivision detected, discuss evolutionary processes that might have accounted for these patterns, and consider conservation management implications for this threatened freshwater fish species.

### Genetic diversity and population structure

Overall *N. obscura* exhibits very low genetic diversity [66], even when compared to other Australian freshwater fishes (mean H_O_ = 0.32 compared to golden perch H_O_ = 0.52 [67], dwarf galaxias H_O_ = 0.40 [68], southern pygmy perch H_O_ = 0.57 [69], and purple spotted gudgeon H_O_ = 0.58 [70]. In addition to overall low levels of diversity, substantial genetic structure was also observed for *N. obscura*. Natural wet and dry cycles, as well as more recent habitat fragmentation have likely resulted in repeated extirpation and re-colonisation events in this species. This boom-bust cycle is thought to account for the small effective population sizes of other freshwater fishes in Australia [71], and accompanied with habitat specificity may be responsible for the low level of genetic diversity observed here. All results of population structure analyses were in agreement with the delineation of the four ESUs proposed by Hammer et al. [32]. Our study builds substantially on the findings of Hammer et al. [32] by the use of highly resolving microsatellite markers, a greater number of sites, and much larger sample size (n = 156, 18 sites Hammer et al. [32], n = 541, 27 sites this study). The high resolution data generated here provide evidence for recent admixture between Merri and Eastern ESUs at site 6 (Curdies River). Mitochondrial DNA reflects evolutionary history [20] and, in this case, the mtDNA gene tree assigns Curdies River to the Eastern ESU [32], a result also supported by allozyme analysis [32]. However, microsatellites depict more recent and fine-scale structure than either mtDNA or allozymes [72]. Conservation management for this site should therefore consider both the historical and geographical connection with Eastern and also seek to maintain contemporary processes responsible for the more recent association with Merri.

Our fine-scale analysis of genetic structure identified 11 hierarchical clusters nested within the four ESUs (Table 1; Fig. 2B). Notably, the Mt Emu and Mustons Creek sites align genetically with Central (to the west of Merri), however they are tributaries of the Hopkins River, which is east of Merri (Fig. 1). Hammer et al. [32] included only Mt Emu Creek in their study and hypothesised an historical connection between the upper Glenelg and Hopkins rivers to explain the anomaly. The inclusion of Mustons Creek in this study and its clear association with Mt Emu Creek and other Central sites supports this hypothesis. An examination of the local topography here reveals a flat region of swampy wetlands extending between the upper reaches of the Glenelg and Hopkins rivers [32]. A similar pattern of closely related populations between these drainages is also found in the sympatric *N. australis* (southern pygmy perch) [73] and it seems very likely that dispersal could occur between these river basins during wetter periods [23], [73]. No genetic structure was detected for the MDB, where the demes appear to be linked by migration. This is probably due to the close proximity of these sites, near contiguous vegetated littoral habitats, and the likelihood of shared refuges during times of drought.

Across the species range, the pairwise F_ST_ estimates of differentiation were mostly significant and high between sites (Table S4), even within clusters. These results highlight the restricted dispersal potential of this species and the isolation by catchment boundaries at many sites. The genetic clusters identified here are consistent across multiple methods of analysis, and satisfy the requirements for designation as MUs [20]. The negligible level of recent migration detected between MUs further supports and validates the other measures of genetic structure.

### Connectivity and the importance of spatial scale

Connectivity is a fundamental ecological and evolutionary process shaping the spatial distribution of genetic variation of species [3]. It is therefore important for conservation management to consider how species biology may alter the way that connectivity is affected by processes such as habitat fragmentation [74], [75]. *Nannoperca obscura* is known to possess limited dispersal potential at all life stages. Small, isolated populations occur in permanent wetlands, streams and ponds and these habitat patches are rarely connected, providing even less opportunity for dispersal. Consequently, environmental changes that result in habitat fragmentation, such as wetland drainage and increased salinity, are likely to have a high impact on the already-limited ability of this species to re-colonise demes after local extirpations. Other Australian fishes inhabiting similar environments also exhibit similar patterns of genetic structure and face the same conservation issues [68], [69], [76]. For example, another native fish *Galaxiella pusilla* (dwarf galaxias) frequently coexists with *N. obscura*, shares many similar life history traits, including restricted dispersal, and is also threatened by habitat fragmentation [68], [77]. Overall genetic diversity for populations of *G. pusilla* is low and broadly similar to *N. obscura*, and population structure for both species follow similar patterns of distribution among catchments. *Macquaria australasica* (Macquarie perch) are a larger percichthyid that although disperses more readily than *N. obscura*, today is restricted to isolated headwaters and shows reduced connectivity associated with human-induced habitat fragmentation [13]. In contrast, the large-bodied percichthyid *Maccullochella peelii* (Murray cod) inhabits the main river channels and larger tributaries, tends to encounter fewer natural barriers to dispersal and is therefore less affected by habitat fragmentation [78].

Environmental and evolutionary processes affecting populations at a local level may differ from those affecting the same species at a regional level [16], [17]. Spatial analyses revealed strong patterns of IBD and population structure at multiple scales for *N. obscura*. At a regional level there is very strong genetic structure with significant divergence between ESUs (Fig. 2A). Within the Eastern ESU a combination of both hierarchical structure (due to population differences between catchments) and IBD exists, while significant IBD was also detectable at a smaller spatial scale within MUs.

Spatial autocorrelation has been used to examine the scale and extent of IBD in several studies [17]–[19]. By identifying the distance at which samples can be considered genetically and demographically independent, and thus defining the range over which this pattern persists, conservation measures can be designed to ensure maximum genetic diversity is preserved [18], [19]. Sampling regimes for genetic monitoring or future ecological studies can also then be designed more efficiently and with confidence that most genetic diversity will be sampled [18]. In the case of *N. obscura*, genetically similar patches are approximately 80 km in diameter for Eastern and Merri and 115 km for Central. This is consistent with the geographic extent of the MUs identified in this study and the proposal that MUs are confined mostly to single catchments.

### Evolutionary processes

Identifying the spatial patterns and scale of genetic variation can help to ensure conservation management strategies capture the overall diversity of a species [79]. To maintain species persistence in the long term, it is also important to identify and conserve the evolutionary processes responsible for generating genetic diversity [22]. The complex patterns of genetic structure observed in this study appear to operate at a range of spatial scales. Findings of previous studies of Australian freshwater fishes [11], [14], [74], [80] have often reported a general pattern of genetic structure concordant with the stream hierarchy model proposed by Meffe and Vrijenhoek [81]. In this model, it is expected that genetic structure will be distributed according to hierarchical drainage structure, with gene flow primarily occurring within rather than among catchments [81]. The strong correspondence between ESU and catchment boundaries across the entire distribution of *N. obscura* suggests that, at a regional scale, the stream hierarchy model is broadly applicable for this species. The significant patterns of IBD evident within three of the ESUs are also predicted under the stream hierarchy model, further supporting this assessment [81].

At a smaller spatial scale however, hydrology has been subjected to significant anthropogenic modification. For example, an extensive network of flood mitigation drains have been constructed throughout the Millicent Coast region [29]. This alteration to the natural hydrology has drained wetlands, and reduced the incidence of flooding by directing surface water to several new coastal outlets [29], [82]. The natural path of flood waters parallel to the coast has therefore been disrupted and isolated coastal populations are now less likely to be linked by floods [29]. As a result, re-colonisation following local extirpation events is unlikely to occur. In many ways the modified hydrological regime described above simulates the drier climate and reduction in frequency of flooding predicted for the wider southeast coastal region under future climate change scenarios [83], which may have implications for *N. obscura* conservation. Indeed, since samples were collected for this study, several Central ESU populations have come under threat or become extirpated [32], [34]. There is also more recent evidence of an increase in the incidence of hybridisation with the co-distributed *N. australis* at some locations [32] and this may be interpreted as a symptom of populations under environmental stress [84], [85]. For instance, Heath et al. [86] showed that a combination of environmental factors was associated with increased levels of hybridisation between sympatric *Oncorhynchus clarkii clarkii* (coastal cutthroat trout) and *O. mykiss* (rainbow trout). The increase in hybridisation observed for *N. obscura* provides further evidence of the negative effects of habitat fragmentation and degradation in this region.

### Conservation implications

The findings presented here have several direct implications for conservation management of *N. obscura*. Using a combination of spatial and non-spatial, and both individual and population based analyses, we identified 11 MUs confined mostly to individual catchments, within which individuals are largely genetically and demographically independent. Conservation of these units should therefore be managed separately in order to maintain the genetic integrity of populations. The MDB lineage appears to be one highly connected population, indicating that family groups for the breeding program can be formed using any combination of fish from different MDB sites. Also, offspring from the breeding program can therefore be released into any MDB site regardless of the specific MDB site of their parents' origin. This permits greater flexibility in selecting the best habitat for release sites. However, populations from other ESUs should not be mixed with the MDB ESU as, due to their significant genetic divergence, this could lead to a reduction in overall fitness because of issues related to local adaptation, and perhaps to outbreeding depression [79], [87].

In addition to providing information for management of the conservation breeding program, there is potential for the same principles to be used to directly manage fragmented wild populations in other ESUs [79]. Translocation has recently received more attention as a viable tool for managing fragmented populations *in situ* [88], [89]. This method has the advantage of maintaining populations in a natural environment, thereby avoiding the potential for adaptation to captivity [89]. The MUs proposed here define the boundaries within which translocations of *N. obscura* might occur should this be considered as a conservation management option in the future. Translocations have however generally had a low success rate in the past [90]. For instance, first and second generation hybrids of native and mixed source translocated *Cottus cognatus* (slimy sculpin) exhibited reduced fitness in a study of the consequences of freshwater fish translocations in southeast Minnesota [91], highlighting the need for further investigation into the genetic effects of translocations.

Habitat fragmentation has clearly had a major influence on the decline of *N. obscura* across its range. Modifications to natural flow regimes have reduced both the amount of available habitat and population connectivity. Given the patchy distribution and low abundance that characterises *N. obscura*, the low genetic diversity and highly differentiated populations uncovered in this study are not surprising. The limited opportunities for dispersal appear dependent upon intermittent flooding that occasionally connects isolated habitat patches. It is therefore critical for the conservation of *N. obscura* that no further modifications to the natural hydrology of this region are undertaken. Furthermore, it is vital that habitat is protected wherever extant populations persist and, where possible, connectivity between populations within MUs is restored to allow natural evolutionary processes to continue.

## Supporting Information

Table S1 Microsatellite markers (Carvalho et al. 2011) amplified for *Nannoperca obscura*.
(DOCX)

Table S2 Geneclass2% probability of individual population assignment of *Nannoperca obscura*.
(DOCX)

Table S3 Pairwise population F_ST_ and R_ST_ for *Nannoperca obscura*.
(DOCX)

Table S4 Pairwise population F_ST_ and R_ST_ for sites where *Nannoperca obscura* samples were collected in multiple years.
(DOCX)

Table S5 Estimated migration rates (*m*) between Evolutionarily Significant Units (ESUs) and 95% credible intervals (CI) calculated with BayesAss.
(DOCX)

Table S6 Estimated migration rates (*m*) between Management Units (MUs) and 95% credible intervals (CI) calculated with BayesAss.
(DOCX)

Figure S1 **Principal coordinates analysis based on 14 microsatellite loci for** ***Nannoperca obscura*** **individuals from each genetic lineage.** A) pure Eastern. Eigen values for the first and second axes have been plotted, which explain 38% and 23% of the variance, respectively. E1, E2 and E3 refer to genetic clusters identified within this lineage; B) Merri/Curdies. Eigen values for the first and second axes explain 40% and 26% of the variance, respectively. M1, M2, and M3 refer to genetic clusters identified within this lineage; C) Central. Eigen values for the first and second axes explain 34% and 26% of the variance, respectively. C1, C2, C3, and C4 refer to genetic clusters identified within this lineage; D) Murray-Darling basin. Eigen values for the first and second axes explain 24% and 22% of the variance, respectively. No genetic structure was apparent within this ESU.
(DOCX)

Figure S2 **Correlograms showing the autocorrelation coefficient** ***r*** **as a function of increasing distance classes for A) pure Eastern, and B) Merri/Curdies ESUs.** Distances are the maximum for each class, grey bars indicate 95% CI about the null hypothesis of no genetic structure, and error bars about r indicate 95% CI as determined by bootstrapping.
(DOCX)

Figure S3 **Correlogram showing the autocorrelation coefficient** ***r*** **as a function of increasing distance classes for Central ESU.** Distances are the maximum for each class, grey bars indicate 95% CI about the null hypothesis of no genetic structure and error bars about *r* indicate 95% CI as determined by bootstrapping.
(DOCX)
