# Supplementary material for: Catchment-Scale Conservation Units Identified for the Threatened Yarra Pygmy Perch (Nannoperca obscura) in Highly Modified River Systems
Source: PLoS One. 2013 Dec 13;8(12):e82953. doi: 10.1371/journal.pone.0082953 (PMC3862729; doi:10.1371/journal.pone.0082953)
Supplement: Table S1 — Microsatellite markers (Carvalho et al. 2011) amplified for Nannoperca obscura. (DOCX) [file pone.0082953.s001.docx]

**Table S1. Microsatellite markers (Carvalho et al. 2011) amplified for *Nannoperca obscura*.**

| Plex | Primer name | Motif | Dye | Forward (5' to 3') | Reverse (5' to 3') |
| --- | --- | --- | --- | --- | --- |
| A | Nob 02 | (TG) 17 | FAM | GTTTAGGCGAGAGGCAAGC | CCGAGCCTGAACCAAGAGG |
| A | Nob 30 | (CA) 14 | VIC | AGGTGGACTGCCTGGCTAAC | CTGTCTGCTGGTGGGTCAC |
| A | Nob 39 | (TG) 10 | VIC | ATGTGAAGGACAGGGTGGAC | AAGATTACAATAAAATTGGTGCTCAG |
| A | Nob 12 | (CA) 16 | NED | CGAGGCGAATAACACTGATGG | AGTGGCCGCATGTATATTGAAC |
| A | Nob 17 | (GT) 16 | PET | TGTTTCGTCCTCAGGGAGC | TCCTTACGTCCATGTTGCAG |
| A | Nob 37 | (TG) 11 | PET | TCATGTTGTTGACCCTCCTG | CCCTCTCCTCTCCTGTTTCC |
| B | Nob 35 | (CA) 13 | FAM | ACTAGGTATCAATAACAGAGGAATGAC | CGTAATAATCACAGCCCTGTTACC |
| B | Nob 16 | (GT) 14 | FAM | CTGCATCGAGCCAGAACTC | CAGCCAGCAGCTCAAATGG |
| B | Nob 36 | (GTT) 7 | VIC | TCCACGATGTTGCACCTAAC | GCCTTGGTTCTCCTCGTTTAG |
| B | Nob 09 | (CA) 15 | VIC | CCTCCTCTGACAACTCCCG | AGGTAGGAAGCAGCTGTGG |
| B | Nob 20 | (CTG) 9 | NED | TCCGTTAGCCATGATCCCG | AAGATGCAGTTCCAGTCCG |
| B | Nob 34 | (CA) 11 | NED | TGTGGTTTGGGACAATAAGCC | GGAGCGATGTTGTGAGATCC |
| B | Nob 32 | (GT) 8 | PET | GTTCAATGCCCTCTTCCAGC | GGTTCTCGCAGCGGTTATC |
| B | Nob 26 | (AGAT) 12 | VIC | GAGGGTCCTGAAGTGGAGC | GCCGTTGCTTCAAATTACCG |
|  |  |  |  |  |  |
