# Supplementary material for: Catchment-Scale Conservation Units Identified for the Threatened Yarra Pygmy Perch (Nannoperca obscura) in Highly Modified River Systems
Source: PLoS One. 2013 Dec 13;8(12):e82953. doi: 10.1371/journal.pone.0082953 (PMC3862729; doi:10.1371/journal.pone.0082953)
Supplement: Table S2 — Geneclass2% probability of individual population assignment of Nannoperca obscura. (DOCX) [file pone.0082953.s002.docx]

**Table S2. Geneclass2 % probability of individual population assignment of *Nannoperca obscura*.**

| ESU | Site |  | 1 | 2 | 3 | 4 | 5 | 6 | 7 | 8 | 9 | 10 | 11 | 12 | 13 | 14 | 15 | 16 | 17 | 18 | 19 | 20 | 21 | 22 | 23 | 24 | 25 | 26 | 27 |
| --- | --- | --- | --- | --- | --- | --- | --- | --- | --- | --- | --- | --- | --- | --- | --- | --- | --- | --- | --- | --- | --- | --- | --- | --- | --- | --- | --- | --- | --- |
| Eastern | 1 | Assigned | 100 | 0 | 0 | 0 | 0 | 0 | 0 | 0 | 0 | 0 | 0 | 0 | 0 | 0 | 0 | 0 | 0 | 0 | 0 | 0 | 0 | 0 | 0 | 0 | 0 | 0 | 0 |
|  |  | Not assigned | 0 |  |  |  |  |  |  |  |  |  |  |  |  |  |  |  |  |  |  |  |  |  |  |  |  |  |  |
|  | 2 | Assigned | 0 | 100 | 12 | 0 | 0 | 0 | 0 | 0 | 0 | 0 | 0 | 0 | 0 | 0 | 0 | 0 | 0 | 0 | 0 | 0 | 0 | 0 | 0 | 0 | 0 | 0 | 0 |
|  |  | Not assigned |  | 0 |  |  |  |  |  |  |  |  |  |  |  |  |  |  |  |  |  |  |  |  |  |  |  |  |  |
|  | 3 | Assigned | 0 | 70 | 90 | 0 | 0 | 0 | 0 | 0 | 0 | 0 | 0 | 0 | 0 | 0 | 0 | 0 | 0 | 0 | 0 | 0 | 0 | 0 | 0 | 0 | 0 | 0 | 0 |
|  |  | Not assigned |  |  | 0 |  |  |  |  |  |  |  |  |  |  |  |  |  |  |  |  |  |  |  |  |  |  |  |  |
|  | 4 | Assigned | 0 | 43 | 0 | 100 | 0 | 0 | 0 | 0 | 0 | 0 | 0 | 0 | 0 | 0 | 0 | 0 | 0 | 0 | 0 | 0 | 0 | 0 | 0 | 0 | 0 | 0 | 0 |
|  |  | Not assigned |  |  |  | 0 |  |  |  |  |  |  |  |  |  |  |  |  |  |  |  |  |  |  |  |  |  |  |  |
|  | 5 | Assigned | 0 | 79 | 10 | 31 | 90 | 0 | 0 | 0 | 0 | 0 | 0 | 0 | 0 | 0 | 0 | 0 | 0 | 0 | 0 | 0 | 0 | 0 | 0 | 0 | 0 | 0 | 0 |
|  |  | Not assigned |  |  |  |  | 3 |  |  |  |  |  |  |  |  |  |  |  |  |  |  |  |  |  |  |  |  |  |  |
| Merri | 6 | Assigned | 0 | 0 | 0 | 0 | 0 | 100 | 0 | 0 | 0 | 7 | 0 | 0 | 0 | 0 | 0 | 0 | 0 | 0 | 0 | 0 | 0 | 0 | 0 | 0 | 0 | 0 | 0 |
|  |  | Not assigned |  |  |  |  |  | 0 |  |  |  |  |  |  |  |  |  |  |  |  |  |  |  |  |  |  |  |  |  |
|  | 7 | Assigned | 0 | 0 | 0 | 0 | 0 | 0 | 97 | 5 | 0 | 13 | 0 | 0 | 0 | 0 | 0 | 0 | 0 | 0 | 0 | 0 | 0 | 0 | 0 | 0 | 0 | 0 | 0 |
|  |  | Not assigned |  |  |  |  |  |  | 3 |  |  |  |  |  |  |  |  |  |  |  |  |  |  |  |  |  |  |  |  |
|  | 8 | Assigned | 0 | 0 | 0 | 0 | 0 | 0 | 0 | 95 | 20 | 68 | 0 | 0 | 0 | 0 | 0 | 0 | 0 | 0 | 0 | 0 | 0 | 0 | 0 | 0 | 0 | 0 | 0 |
|  |  | Not assigned |  |  |  |  |  |  |  | 3 |  |  |  |  |  |  |  |  |  |  |  |  |  |  |  |  |  |  |  |
|  | 9 | Assigned | 0 | 0 | 0 | 0 | 0 | 0 | 0 | 30 | 93 | 100 | 0 | 0 | 0 | 0 | 0 | 0 | 0 | 0 | 0 | 0 | 0 | 0 | 0 | 0 | 0 | 0 | 0 |
|  |  | Not assigned |  |  |  |  |  |  |  |  | 0 |  |  |  |  |  |  |  |  |  |  |  |  |  |  |  |  |  |  |
|  | 10 | Assigned | 0 | 0 | 0 | 0 | 0 | 0 | 0 | 25 | 38 | 63 | 0 | 0 | 0 | 0 | 0 | 0 | 0 | 0 | 0 | 0 | 0 | 0 | 0 | 0 | 0 | 0 | 0 |
|  |  | Not assigned |  |  |  |  |  |  |  |  |  | 25 |  |  |  |  |  |  |  |  |  |  |  |  |  |  |  |  |  |
| Central | 11 | Assigned | 0 | 0 | 0 | 0 | 0 | 0 | 0 | 0 | 0 | 6 | 97 | 0 | 0 | 0 | 6 | 6 | 0 | 0 | 0 | 6 | 10 | 0 | 0 | 0 | 0 | 0 | 0 |
|  |  | Not assigned |  |  |  |  |  |  |  |  |  |  | 3 |  |  |  |  |  |  |  |  |  |  |  |  |  |  |  |  |
|  | 12 | Assigned | 0 | 0 | 0 | 0 | 0 | 0 | 0 | 0 | 0 | 0 | 0 | 90 | 0 | 0 | 0 | 60 | 0 | 0 | 5 | 15 | 0 | 5 | 0 | 0 | 0 | 0 | 0 |
|  |  | Not assigned |  |  |  |  |  |  |  |  |  |  |  | 10 |  |  |  |  |  |  |  |  |  |  |  |  |  |  |  |
|  | 13 | Assigned | 0 | 0 | 0 | 0 | 0 | 0 | 0 | 0 | 0 | 3 | 0 | 0 | 100 | 68 | 0 | 0 | 0 | 0 | 0 | 0 | 0 | 0 | 0 | 0 | 0 | 0 | 0 |
|  |  | Not assigned |  |  |  |  |  |  |  |  |  |  |  |  | 0 |  |  |  |  |  |  |  |  |  |  |  |  |  |  |
|  | 14 | Assigned | 0 | 0 | 0 | 0 | 0 | 0 | 0 | 0 | 0 | 0 | 0 | 0 | 0 | 75 | 0 | 0 | 0 | 0 | 0 | 0 | 0 | 0 | 0 | 0 | 0 | 0 | 0 |
|  |  | Not assigned |  |  |  |  |  |  |  |  |  |  |  |  |  | 25 |  |  |  |  |  |  |  |  |  |  |  |  |  |
|  | 15 | Assigned | 0 | 0 | 0 | 0 | 0 | 0 | 0 | 0 | 0 | 0 | 0 | 0 | 0 | 0 | 60 | 70 | 0 | 30 | 10 | 10 | 30 | 20 | 0 | 0 | 0 | 0 | 0 |
|  |  | Not assigned |  |  |  |  |  |  |  |  |  |  |  |  |  |  | 20 |  |  |  |  |  |  |  |  |  |  |  |  |
|  | 16 | Assigned | 0 | 0 | 0 | 0 | 0 | 0 | 0 | 0 | 0 | 0 | 0 | 0 | 0 | 0 | 80 | 90 | 0 | 0 | 30 | 50 | 30 | 30 | 0 | 0 | 0 | 0 | 0 |
|  |  | Not assigned |  |  |  |  |  |  |  |  |  |  |  |  |  |  |  | 10 |  |  |  |  |  |  |  |  |  |  |  |
|  | 17 | Assigned | 0 | 0 | 0 | 0 | 0 | 0 | 0 | 0 | 0 | 15 | 9 | 0 | 0 | 0 | 68 | 56 | 88 | 94 | 79 | 88 | 94 | 88 | 0 | 0 | 0 | 0 | 0 |
|  |  | Not assigned |  |  |  |  |  |  |  |  |  |  |  |  |  |  |  |  | 0 |  |  |  |  |  |  |  |  |  |  |
|  | 18 | Assigned | 0 | 0 | 0 | 0 | 0 | 0 | 0 | 0 | 0 | 0 | 0 | 0 | 0 | 0 | 80 | 60 | 80 | 80 | 70 | 70 | 100 | 80 | 0 | 0 | 0 | 0 | 0 |
|  |  | Not assigned |  |  |  |  |  |  |  |  |  |  |  |  |  |  |  |  |  | 0 |  |  |  |  |  |  |  |  |  |
|  | 19 | Assigned | 0 | 0 | 0 | 0 | 0 | 0 | 0 | 0 | 0 | 11 | 11 | 0 | 0 | 0 | 67 | 89 | 56 | 67 | 89 | 78 | 100 | 100 | 0 | 0 | 0 | 0 | 0 |
|  |  | Not assigned |  |  |  |  |  |  |  |  |  |  |  |  |  |  |  |  |  |  | 0 |  |  |  |  |  |  |  |  |
|  | 20 | Assigned | 0 | 0 | 0 | 0 | 0 | 0 | 0 | 0 | 0 | 0 | 0 | 0 | 0 | 0 | 25 | 38 | 25 | 31 | 56 | 94 | 50 | 75 | 0 | 0 | 0 | 0 | 0 |
|  |  | Not assigned |  |  |  |  |  |  |  |  |  |  |  |  |  |  |  |  |  |  |  | 6 |  |  |  |  |  |  |  |
|  | 21 | Assigned | 0 | 0 | 0 | 0 | 0 | 0 | 0 | 0 | 0 | 14 | 7 | 0 | 0 | 0 | 71 | 79 | 79 | 79 | 86 | 86 | 86 | 93 | 0 | 0 | 0 | 0 | 0 |
|  |  | Not assigned |  |  |  |  |  |  |  |  |  |  |  |  |  |  |  |  |  |  |  |  | 7 |  |  |  |  |  |  |
|  | 22 | Assigned | 0 | 0 | 0 | 0 | 0 | 0 | 0 | 0 | 0 | 0 | 0 | 0 | 0 | 0 | 69 | 77 | 50 | 58 | 77 | 81 | 88 | 92 | 0 | 0 | 0 | 0 | 0 |
|  |  | Not assigned |  |  |  |  |  |  |  |  |  |  |  |  |  |  |  |  |  |  |  |  |  | 4 |  |  |  |  |  |
| MDB | 23 | Assigned | 0 | 0 | 0 | 0 | 0 | 0 | 0 | 0 | 0 | 0 | 0 | 0 | 0 | 0 | 0 | 0 | 0 | 0 | 0 | 0 | 0 | 0 | 80 | 60 | 80 | 80 | 80 |
|  |  | Not assigned |  |  |  |  |  |  |  |  |  |  |  |  |  |  |  |  |  |  |  |  |  |  | 20 |  |  |  |  |
|  | 24 | Assigned | 0 | 0 | 0 | 0 | 0 | 0 | 0 | 0 | 0 | 0 | 0 | 0 | 0 | 0 | 0 | 0 | 0 | 0 | 0 | 0 | 0 | 0 | 100 | 80 | 100 | 100 | 100 |
|  |  | Not assigned |  |  |  |  |  |  |  |  |  |  |  |  |  |  |  |  |  |  |  |  |  |  |  | 0 |  |  |  |
|  | 25 | Assigned | 0 | 0 | 0 | 0 | 0 | 0 | 0 | 0 | 0 | 0 | 0 | 0 | 0 | 0 | 0 | 0 | 0 | 0 | 0 | 0 | 0 | 0 | 65 | 65 | 88 | 92 | 88 |
|  |  | Not assigned |  |  |  |  |  |  |  |  |  |  |  |  |  |  |  |  |  |  |  |  |  |  |  |  | 0 |  |  |
|  | 26 | Assigned | 0 | 0 | 0 | 0 | 0 | 0 | 0 | 0 | 0 | 0 | 0 | 0 | 0 | 0 | 0 | 0 | 0 | 0 | 0 | 0 | 0 | 0 | 47 | 82 | 94 | 88 | 100 |
|  |  | Not assigned |  |  |  |  |  |  |  |  |  |  |  |  |  |  |  |  |  |  |  |  |  |  |  |  |  | 0 |  |
|  | 27 | Assigned | 0 | 0 | 0 | 0 | 0 | 0 | 0 | 0 | 0 | 0 | 0 | 0 | 0 | 0 | 0 | 0 | 0 | 0 | 0 | 0 | 0 | 0 | 53 | 56 | 97 | 97 | 97 |
|  |  | Not assigned |  |  |  |  |  |  |  |  |  |  |  |  |  |  |  |  |  |  |  |  |  |  |  |  |  |  | 3 |

Population assignment was calculated separately for each individual and each population, therefore assignment totals may exceed 100% as some individuals have >5% probability for more than one population.
