# Supplementary material for: Catchment-Scale Conservation Units Identified for the Threatened Yarra Pygmy Perch (Nannoperca obscura) in Highly Modified River Systems
Source: PLoS One. 2013 Dec 13;8(12):e82953. doi: 10.1371/journal.pone.0082953 (PMC3862729; doi:10.1371/journal.pone.0082953)
Supplement: Table S3 — Pairwise population FST and RST for Nannoperca obscura. (DOCX) [file pone.0082953.s003.docx]

**Table S3. Pairwise population F_ST_ and R_ST_ (above and below the diagonal, respectively) for *Nannoperca obscura*.**

| Population | 1 | 2 | 3 | 4 | 5 | 6 | 7 | 8 | 9 | 10 | 11 | 12 | 13 | 14 | 15 | 16 | 17 | 18 | 19 | 20 | 21 | 22 | 23 | 24 | 25 | 26 | 27 |
| --- | --- | --- | --- | --- | --- | --- | --- | --- | --- | --- | --- | --- | --- | --- | --- | --- | --- | --- | --- | --- | --- | --- | --- | --- | --- | --- | --- |
| 1 | **–** | **0.35** | **0.58** | **0.55** | **0.58** | **0.68** | **0.53** | **0.60** | **0.55** | **0.59** | **0.63** | **0.84** | **0.72** | **0.66** | **0.65** | **0.64** | **0.65** | **0.68** | **0.71** | **0.64** | **0.69** | **0.63** | **0.72** | **0.76** | **0.63** | **0.67** | **0.65** |
| 2 | **0.36** | **–** | **0.15** | **0.14** | **0.22** | **0.38** | **0.36** | **0.39** | **0.33** | **0.29** | **0.41** | **0.55** | **0.48** | **0.36** | **0.39** | **0.39** | **0.44** | **0.40** | **0.41** | **0.38** | **0.40** | **0.41** | **0.46** | **0.46** | **0.48** | **0.46** | **0.49** |
| 3 | **0.51** | **0.15** | **–** | **0.23** | **0.35** | **0.40** | **0.41** | **0.40** | **0.37** | **0.36** | **0.50** | **0.68** | **0.61** | **0.46** | **0.50** | **0.48** | **0.54** | **0.52** | **0.53** | **0.48** | **0.52** | **0.51** | **0.56** | **0.58** | **0.58** | **0.57** | **0.58** |
| 4 | **0.48** | **0.27** | 0.09 | **–** | **0.19** | **0.50** | **0.47** | **0.50** | **0.45** | **0.43** | **0.55** | **0.73** | **0.65** | **0.50** | **0.51** | **0.50** | **0.59** | **0.57** | **0.58** | **0.53** | **0.57** | **0.54** | **0.56** | **0.58** | **0.57** | **0.57** | **0.58** |
| 5 | **0.47** | **0.49** | **0.33** | **0.18** | **–** | **0.55** | **0.58** | **0.60** | **0.55** | **0.56** | **0.63** | **0.73** | **0.68** | **0.61** | **0.62** | **0.61** | **0.64** | **0.64** | **0.65** | **0.61** | **0.64** | **0.62** | **0.66** | **0.67** | **0.64** | **0.65** | **0.65** |
| 6 | **0.88** | **0.81** | **0.80** | **0.79** | **0.49** | **–** | **0.42** | **0.41** | **0.44** | **0.44** | **0.60** | **0.72** | **0.67** | **0.61** | **0.63** | **0.61** | **0.62** | **0.64** | **0.63** | **0.60** | **0.62** | **0.60** | **0.68** | **0.69** | **0.65** | **0.66** | **0.65** |
| 7 | **0.30** | **0.31** | **0.17** | **0.17** | **0.35** | **0.66** | **–** | **0.24** | **0.28** | **0.28** | **0.48** | **0.64** | **0.56** | **0.47** | **0.49** | **0.46** | **0.47** | **0.48** | **0.48** | **0.46** | **0.48** | **0.48** | **0.57** | **0.58** | **0.57** | **0.57** | **0.58** |
| 8 | **0.25** | **0.29** | **0.10** | **0.09** | **0.28** | **0.57** | **0.06** | **–** | **0.09** | **0.12** | **0.42** | **0.61** | **0.46** | **0.39** | **0.47** | **0.43** | **0.43** | **0.43** | **0.43** | **0.42** | **0.43** | **0.43** | **0.57** | **0.57** | **0.56** | **0.56** | **0.57** |
| 9 | **0.34** | **0.35** | **0.14** | **0.14** | **0.31** | **0.56** | **0.14** | **0.04** | **–** | **0.05** | **0.31** | **0.54** | **0.38** | **0.29** | **0.37** | **0.33** | **0.32** | **0.33** | **0.31** | **0.31** | **0.31** | **0.32** | **0.52** | **0.51** | **0.51** | **0.50** | **0.52** |
| 10 | **0.38** | **0.29** | 0.10 | 0.11 | **0.34** | **0.74** | **0.14** | 0.04 | 0.01 | **–** | **0.35** | **0.63** | **0.45** | **0.31** | **0.39** | **0.36** | **0.37** | **0.37** | **0.36** | **0.34** | **0.36** | **0.36** | **0.52** | **0.52** | **0.52** | **0.52** | **0.53** |
| 11 | **0.60** | **0.60** | **0.43** | **0.38** | **0.37** | **0.58** | **0.37** | **0.26** | **0.18** | **0.27** | **–** | **0.52** | **0.44** | **0.25** | **0.25** | **0.22** | **0.22** | **0.23** | **0.21** | **0.20** | **0.22** | **0.19** | **0.53** | **0.52** | **0.49** | **0.48** | **0.48** |
| 12 | **0.93** | **0.77** | **0.78** | **0.77** | **0.43** | **0.83** | **0.47** | **0.42** | **0.43** | **0.66** | **0.37** | **–** | **0.66** | **0.58** | **0.52** | **0.42** | **0.56** | **0.62** | **0.55** | **0.49** | **0.60** | **0.47** | **0.72** | **0.73** | **0.63** | **0.65** | **0.62** |
| 13 | **0.92** | **0.83** | **0.82** | **0.84** | **0.70** | **0.84** | **0.60** | **0.51** | **0.40** | **0.67** | **0.36** | **0.86** | **–** | **0.24** | **0.47** | **0.45** | **0.46** | **0.50** | **0.50** | **0.43** | **0.51** | **0.44** | **0.67** | **0.67** | **0.62** | **0.63** | **0.62** |
| 14 | **0.79** | **0.70** | **0.57** | **0.56** | **0.49** | **0.76** | **0.35** | **0.28** | **0.19** | **0.35** | **0.14** | **0.62** | **0.29** | **–** | **0.28** | **0.27** | **0.32** | **0.33** | **0.35** | **0.29** | **0.34** | **0.29** | **0.51** | **0.52** | **0.50** | **0.50** | **0.50** |
| 15 | **0.82** | **0.68** | **0.57** | **0.53** | **0.34** | **0.68** | **0.41** | **0.29** | **0.20** | **0.37** | **0.05** | **0.62** | **0.62** | **0.30** | **–** | 0.03 | **0.16** | **0.11** | **0.13** | **0.20** | **0.16** | **0.10** | **0.46** | **0.44** | **0.41** | **0.39** | **0.40** |
| 16 | **0.78** | **0.66** | **0.54** | **0.48** | **0.29** | **0.67** | **0.41** | **0.29** | **0.24** | **0.38** | **0.08** | **0.50** | **0.68** | **0.34** | 0.00 | **–** | **0.17** | **0.14** | **0.11** | **0.17** | **0.16** | **0.09** | **0.44** | **0.43** | **0.41** | **0.38** | **0.41** |
| 17 | **0.84** | **0.74** | **0.68** | **0.67** | **0.49** | **0.71** | **0.53** | **0.40** | **0.28** | **0.51** | **0.14** | **0.74** | **0.57** | **0.47** | **0.14** | **0.30** | **–** | 0.01 | **0.05** | **0.12** | **0.03** | **0.07** | **0.57** | **0.56** | **0.52** | **0.51** | **0.51** |
| 18 | **0.87** | **0.70** | **0.62** | **0.62** | **0.40** | **0.72** | **0.46** | **0.33** | **0.22** | **0.41** | **0.12** | **0.80** | **0.66** | **0.44** | **0.11** | **0.24** | 0.00 | **–** | **0.06** | **0.14** | 0.04 | **0.08** | **0.54** | **0.53** | **0.48** | **0.47** | **0.48** |
| 19 | **0.84** | **0.68** | **0.59** | **0.57** | **0.35** | **0.69** | **0.45** | **0.32** | **0.22** | **0.38** | **0.09** | **0.74** | **0.65** | **0.40** | 0.03 | **0.12** | 0.03 | 0.00 | **–** | **0.06** | 0.03 | 0.02 | **0.54** | **0.53** | **0.47** | **0.47** | **0.47** |
| 20 | **0.77** | **0.69** | **0.59** | **0.54** | **0.38** | **0.60** | **0.48** | **0.35** | **0.24** | **0.39** | **0.14** | **0.65** | **0.52** | **0.36** | **0.11** | **0.21** | **0.05** | **0.06** | 0.03 | **–** | **0.14** | **0.11** | **0.52** | **0.52** | **0.49** | **0.48** | **0.49** |
| 21 | **0.84** | **0.69** | **0.61** | **0.59** | **0.38** | **0.71** | **0.46** | **0.34** | **0.23** | **0.42** | **0.10** | **0.73** | **0.64** | **0.42** | 0.05 | **0.16** | 0.03 | 0.00 | 0.00 | **0.05** | **–** | **0.03** | **0.55** | **0.54** | **0.48** | **0.47** | **0.47** |
| 22 | **0.77** | **0.69** | **0.58** | **0.54** | **0.37** | **0.65** | **0.47** | **0.36** | **0.28** | **0.44** | **0.11** | **0.53** | **0.59** | **0.37** | 0.00 | 0.05 | **0.13** | **0.09** | 0.02 | **0.11** | 0.04 | **–** | **0.51** | **0.49** | **0.45** | **0.43** | **0.44** |
| 23 | **0.80** | **0.76** | **0.67** | **0.65** | **0.61** | **0.86** | **0.49** | **0.48** | **0.51** | **0.58** | **0.55** | **0.72** | **0.85** | **0.50** | **0.66** | **0.61** | **0.82** | **0.76** | **0.72** | **0.73** | **0.75** | **0.71** | **–** | **0.07** | **0.06** | 0.03 | **0.12** |
| 24 | **0.95** | **0.76** | **0.74** | **0.76** | **0.58** | **0.89** | **0.44** | **0.40** | **0.44** | **0.61** | **0.47** | **0.87** | **0.89** | **0.57** | **0.73** | **0.66** | **0.83** | **0.86** | **0.81** | **0.76** | **0.82** | **0.71** | 0.03 | **–** | 0.02 | 0.01 | **0.05** |
| 25 | **0.64** | **0.70** | **0.58** | **0.55** | **0.57** | **0.74** | **0.47** | **0.46** | **0.50** | **0.55** | **0.49** | **0.47** | **0.69** | **0.42** | **0.52** | **0.48** | **0.69** | **0.61** | **0.58** | **0.63** | **0.61** | **0.59** | 0.00 | 0.00 | **–** | 0.00 | **0.02** |
| 26 | **0.93** | **0.74** | **0.73** | **0.77** | **0.56** | **0.87** | **0.40** | **0.31** | **0.36** | **0.61** | **0.37** | **0.86** | **0.88** | **0.66** | **0.69** | **0.56** | **0.76** | **0.81** | **0.76** | **0.71** | **0.76** | **0.63** | 0.32 | **0.00** | 0.00 | **–** | 0.00 |
| 27 | **0.71** | **0.75** | **0.66** | **0.63** | **0.63** | **0.79** | **0.53** | **0.52** | **0.56** | **0.63** | **0.56** | **0.55** | **0.74** | **0.50** | **0.61** | **0.57** | **0.74** | **0.69** | **0.66** | **0.69** | **0.68** | **0.66** | 0.00 | 0.05 | 0.00 | 0.00 | **–** |

Significant values (P<0.05) are indicated in bold.
