# Supplementary material for: Catchment-Scale Conservation Units Identified for the Threatened Yarra Pygmy Perch (Nannoperca obscura) in Highly Modified River Systems
Source: PLoS One. 2013 Dec 13;8(12):e82953. doi: 10.1371/journal.pone.0082953 (PMC3862729; doi:10.1371/journal.pone.0082953)
Supplement: Table S4 — Pairwise population FST and RST for sites where Nannoperca obscura samples were collected in multiple years. (DOCX) [file pone.0082953.s004.docx]

**Table S4. Pairwise population F_ST_ and R_ST_ for sites where *Nannoperca obscura* samples were collected in multiple years.**

| Site | Years sampled | F_ST_ | P value | R_ST_ | P value |
| --- | --- | --- | --- | --- | --- |
| 2 | 2000, 2002 | 0.04 | **0.003** | 0.03 | 0.056 |
| 4 | 2000, 2003 | 0.07 | 0.141 | 0.00 | 0.567 |
| 6 | 2000, 2002 | 0.01 | 0.306 | 0.02 | 0.162 |
| 7 | 2000, 2002 | 0.01 | 0.211 | 0.01 | 0.216 |
| 8 | 2000, 2002 | 0.00 | 0.737 | 0.00 | 0.342 |
| 11 | 2000, 2002 | 0.01 | 0.175 | 0.00 | 0.325 |
| 12 | 2004, 2011 | 0.02 | 0.264 | 0.12 | 0.069 |
| 13 | 2000, 2002 | 0.03 | 0.100 | 0.01 | 0.213 |
| 19 | 2004, 2009 | 0.00 | 1.000 | 0.16 | 0.999 |
| 20 | 2001, 2010 | 0.00 | 0.318 | 0.02 | 0.271 |
| 23 | 2001, 2002 | 0.01 | 0.495 | 0.03 | 0.900 |
| 25 | 2004, 2007 | 0.00 | 0.745 | 0.00 | 0.994 |

Significant value indicated in bold.
