# Supplementary material for: Catchment-Scale Conservation Units Identified for the Threatened Yarra Pygmy Perch (Nannoperca obscura) in Highly Modified River Systems
Source: PLoS One. 2013 Dec 13;8(12):e82953. doi: 10.1371/journal.pone.0082953 (PMC3862729; doi:10.1371/journal.pone.0082953)
Supplement: Table S5 — Estimated migration rates (m) between Evolutionarily Significant Units (ESUs) and 95% credible intervals (CI) calculated with BayesAss. (DOCX) [file pone.0082953.s005.docx]

**Table S5. Estimated migration rates (*m*) between Evolutionarily Significant Units (ESUs) and 95% credible intervals (CI) calculated with BayesAss.**

| From | To | *m* | 95% CI |
| --- | --- | --- | --- |
| Eastern | Merri | 0.004 | (-0.003 – 0.011) |
| Eastern | Central | 0.004 | (-0.003 – 0.010) |
| Eastern | MDB | 0.004 | (-0.003 – 0.011) |
|  |  |  |  |
| Merri | Eastern | 0.003 | (-0.002 – 0.008) |
| Merri | Central | 0.007 | (-0.002 – 0.015) |
| Merri | MDB | 0.002 | (-0.002 – 0.007) |
|  |  |  |  |
| Central | Eastern | 0.002 | (-0.001 – 0.004) |
| Central | Merri | 0.002 | (-0.001 – 0.004) |
| Central | MDB | 0.002 | (-0.001 – 0.004) |
|  |  |  |  |
| MDB | Eastern | 0.004 | (-0.003 – 0.011) |
| MDB | Merri | 0.004 | (-0.004 – 0.011) |
| MDB | Central | 0.004 | (-0.003 – 0.011) |
