# Supplementary material for: Catchment-Scale Conservation Units Identified for the Threatened Yarra Pygmy Perch (Nannoperca obscura) in Highly Modified River Systems
Source: PLoS One. 2013 Dec 13;8(12):e82953. doi: 10.1371/journal.pone.0082953 (PMC3862729; doi:10.1371/journal.pone.0082953)
Supplement: Table S6 — Estimated migration rates (m) between Management Units (MUs) and 95% credible intervals (CI) calculated with BayesAss. (DOCX) [file pone.0082953.s006.docx]

**Table S6. Estimated migration rates (*m*) between Management Units (MUs) and 95% credible intervals (CI) calculated with BayesAss.**

| From | To | *m* | 95% CI |
| --- | --- | --- | --- |
| E1 | E2 | 0.016 | (-0.01 – 0.05) |
| E1 | E3 | 0.016 | (-0.01 – 0.04) |
| E2 | E1 | 0.006 | (-0.01 – 0.02) |
| E2 | E3 | 0.007 | (-0.01 – 0.02) |
| E3 | E1 | 0.007 | (-0.01 – 0.02) |
| E3 | E2 | 0.008 | (-0.01 – 0.02) |
|  |  |  |  |
| M1 | M2 | 0.008 | (-0.01 – 0.02) |
| M1 | M3 | 0.008 | (-0.01 – 0.02) |
| M2 | M1 | 0.007 | (-0.01 – 0.02) |
| M2 | M3 | 0.007 | (-0.01 – 0.02) |
| M3 | M1 | 0.004 | (0.00 – 0.01) |
| M3 | M2 | 0.005 | (0.00 – 0.01) |
|  |  |  |  |
| C1 | C2 | 0.008 | (-0.01 – 0.02) |
| C1 | C3 | 0.008 | (-0.01 – 0.02) |
| C1 | C4 | 0.021 | (-0.02 – 0.06) |
| C2 | C1 | 0.011 | (-0.01 – 0.03) |
| C2 | C3 | 0.011 | (-0.01 – 0.03) |
| C2 | C4 | 0.018 | (-0.02 – 0.05) |
| C3 | C1 | 0.009 | (-0.01 – 0.03) |
| C3 | C2 | 0.007 | (-0.01 – 0.02) |
| C3 | C4 | 0.008 | (-0.01 – 0.02) |
| C4 | C1 | 0.003 | (0.00 – 0.01) |
| C4 | C2 | 0.003 | (0.00 – 0.01) |
| C4 | C3 | 0.002 | (0.00 – 0.01) |
