# Supplementary material for: Catchment-Scale Conservation Units Identified for the Threatened Yarra Pygmy Perch (Nannoperca obscura) in Highly Modified River Systems
Source: PLoS One. 2013 Dec 13;8(12):e82953. doi: 10.1371/journal.pone.0082953 (PMC3862729; doi:10.1371/journal.pone.0082953)
Supplement: Figure S1 — Principal coordinates analysis based on 14 microsatellite loci for Nannoperca obscura individuals from each genetic lineage. A) pure Eastern. Eigen values for the first and second axes have been plotted, which explain 38% and 23% of the variance, respectively. E1, E2 and E3 refer to genetic clusters identified within this lineage; B) Merri/Curdies. Eigen values for the first and second axes explain 40% and 26% of the variance, respectively. M1, M2, and M3 refer to genetic clusters identified within this lineage; C) Central. Eigen values for the first and second axes explain 34% and 26% of the variance, respectively. C1, C2, C3, and C4 refer to genetic clusters identified within this lineage; D) Murray-Darling basin. Eigen values for the first and second axes explain 24% and 22% of the variance, respectively. No genetic structure was apparent within this ESU. (DOCX) [file pone.0082953.s007.docx]

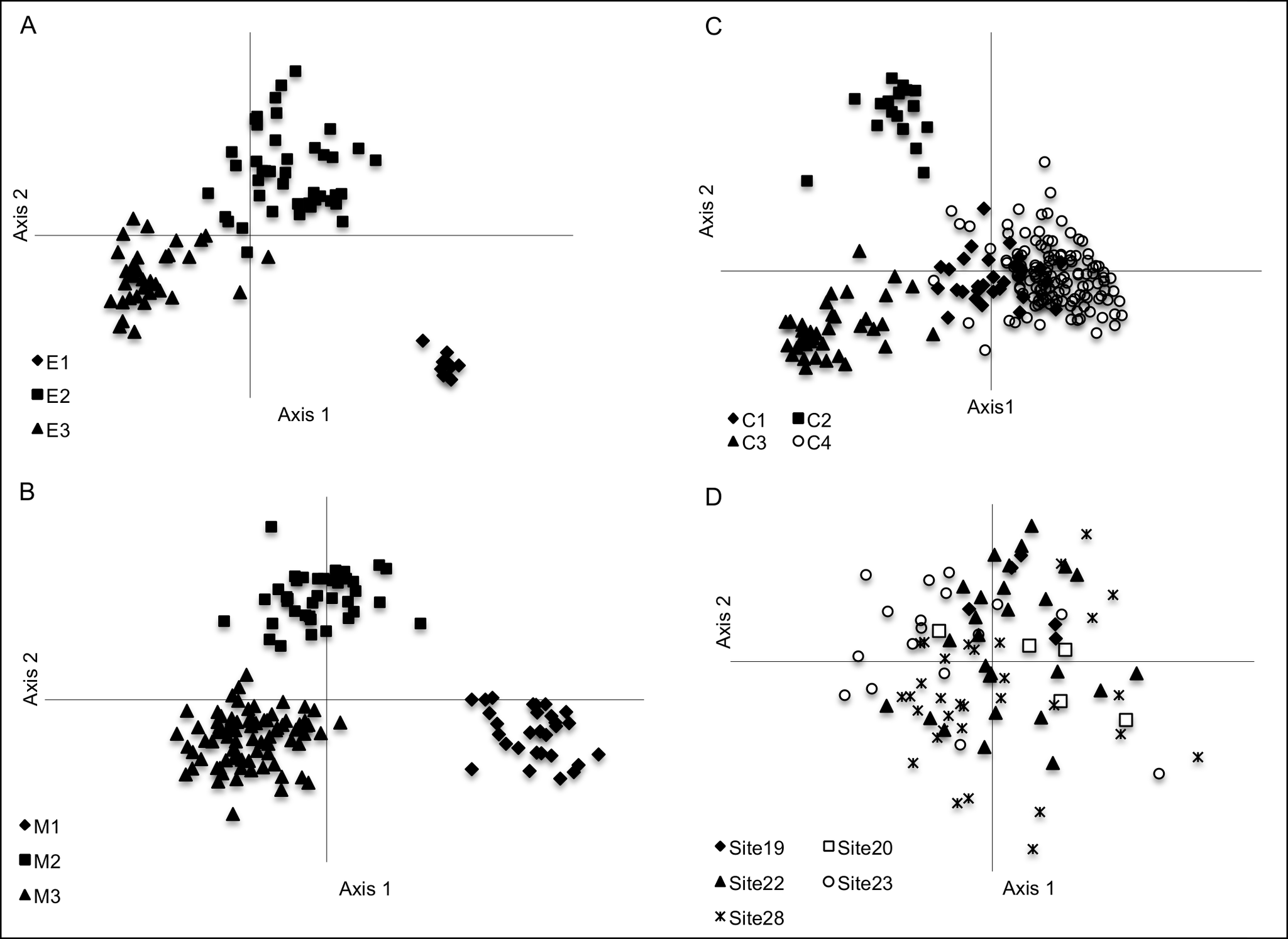


**Figure S1. Principal coordinates analysis based on 14 microsatellite loci for *Nannoperca obscura* individuals from each genetic lineage.** A) pure Eastern. Eigen values for the first and second axes have been plotted, which explain 38% and 23% of the variance, respectively. E1, E2 and E3 refer to genetic clusters identified within this lineage; B) Merri/Curdies. Eigen values for the first and second axes explain 40% and 26% of the variance, respectively. M1, M2, and M3 refer to genetic clusters identified within this lineage; C) Central. Eigen values for the first and second axes explain 34% and 26% of the variance, respectively. C1, C2, C3, and C4 refer to genetic clusters identified within this lineage; D) Murray-Darling basin. Eigen values for the first and second axes explain 24% and 22% of the variance, respectively. No genetic structure was apparent within this ESU
