# Supplementary material for: Catchment-Scale Conservation Units Identified for the Threatened Yarra Pygmy Perch (Nannoperca obscura) in Highly Modified River Systems
Source: PLoS One. 2013 Dec 13;8(12):e82953. doi: 10.1371/journal.pone.0082953 (PMC3862729; doi:10.1371/journal.pone.0082953)
Supplement: Figure S2 — Correlograms showing the autocorrelation coefficient r as a function of increasing distance classes for A) pure Eastern, and B) Merri/Curdies ESUs. Distances are the maximum for each class, grey bars indicate 95% CI about the null hypothesis of no genetic structure, and error bars about r indicate 95% CI as determined by bootstrapping. (DOCX) [file pone.0082953.s008.docx]

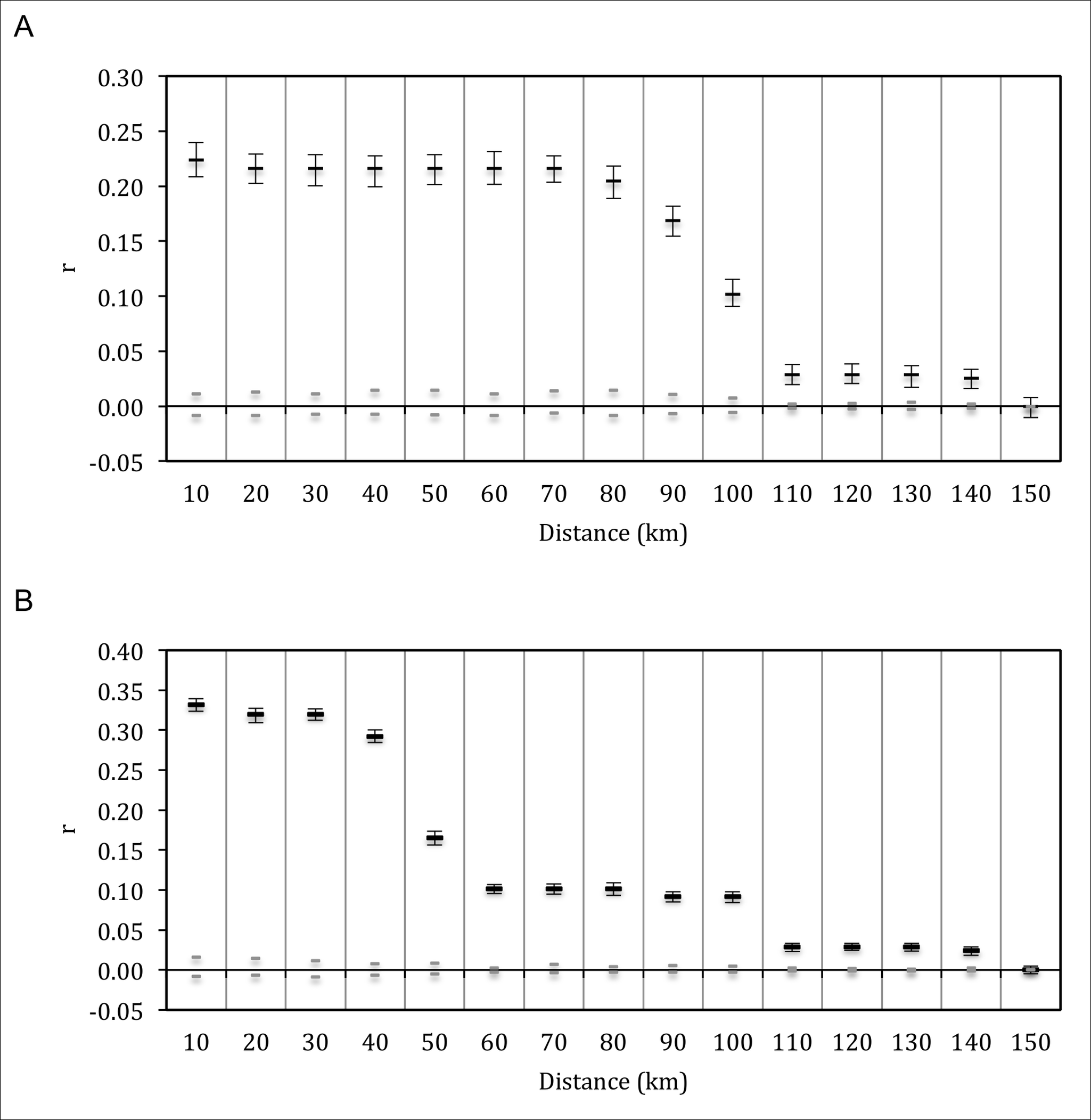


**Figure S2. Correlograms showing the autocorrelation coefficient *r* as a function of increasing distance classes for A) pure Eastern, and B) Merri/Curdies ESUs.** Distances are the maximum for each class, grey bars indicate 95% CI about the null hypothesis of no genetic structure, and error bars about r indicate 95% CI as determined by bootstrapping.
