# Supplementary material for: Catchment-Scale Conservation Units Identified for the Threatened Yarra Pygmy Perch (Nannoperca obscura) in Highly Modified River Systems
Source: PLoS One. 2013 Dec 13;8(12):e82953. doi: 10.1371/journal.pone.0082953 (PMC3862729; doi:10.1371/journal.pone.0082953)
Supplement: Figure S3 — Correlogram showing the autocorrelation coefficient r as a function of increasing distance classes for Central ESU. Distances are the maximum for each class, grey bars indicate 95% CI about the null hypothesis of no genetic structure and error bars about r indicate 95% CI as determined by bootstrapping. (DOCX) [file pone.0082953.s009.docx]

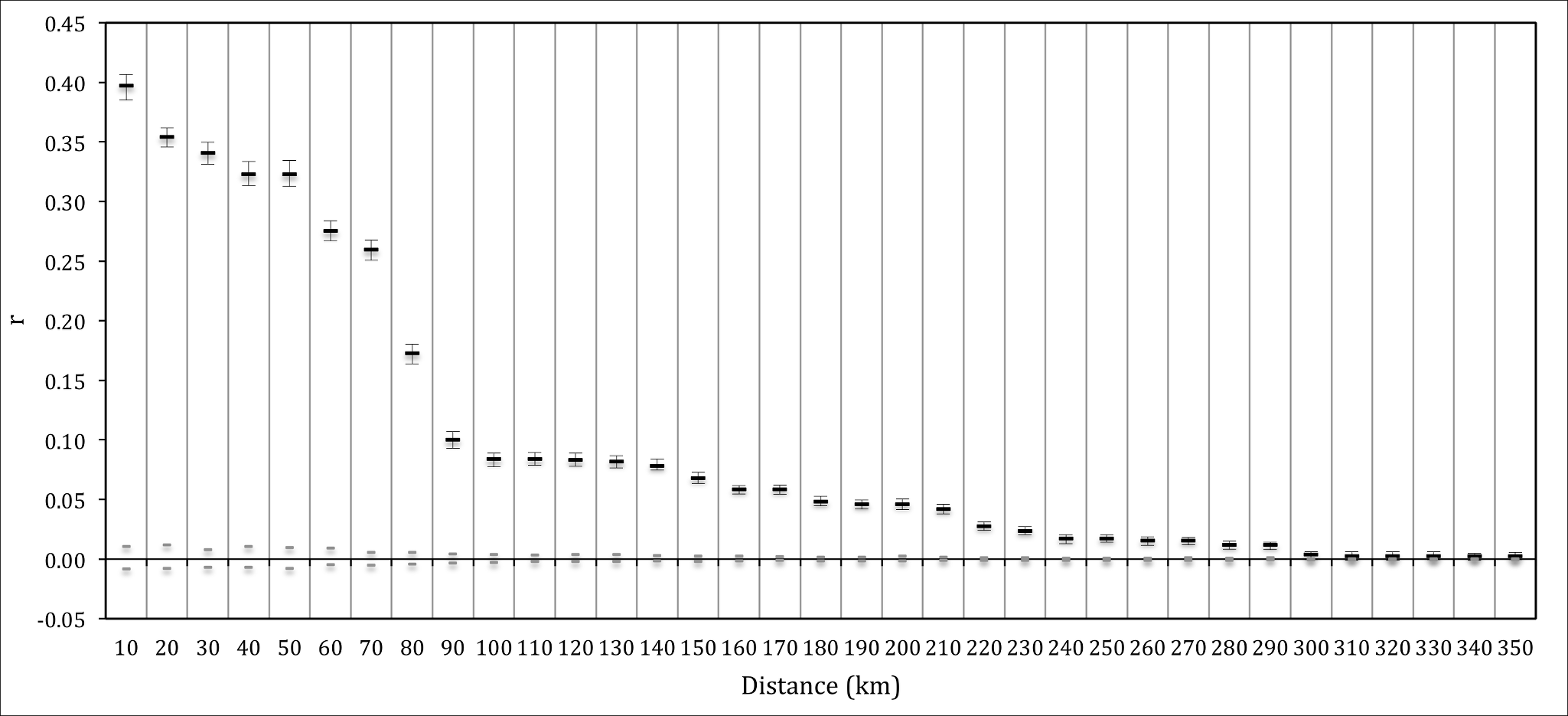


**Figure S3. Correlogram showing the autocorrelation coefficient *r* as a function of increasing distance classes for Central ESU.** Distances are the maximum for each class, grey bars indicate 95% CI about the null hypothesis of no genetic structure and error bars about *r* indicate 95% CI as determined by bootstrapping.
